# Design, Synthesis, and Bioactivity Evaluation of Novel Isoxazole-Amide Derivatives Containing an Acylhydrazone Moiety as New Active Antiviral Agents

**DOI:** 10.3390/molecules24203766

**Published:** 2019-10-19

**Authors:** Zai-Bo Yang, Pei Li, Yin-Ju He

**Affiliations:** 1 School of Chemistry and Chemical Engineering, Qiannan Normal University for Nationalities, Duyun 558000, China; heyinju2007@163.com; 2 Qiandongnan Engineering and Technology Research Center for Comprehensive Utilization of National Medicine, Kaili University, Kaili 556011, China

**Keywords:** isoxazole-amide, acylhydrazone, synthesis, antiviral activity

## Abstract

As a continuation of our efforts to discover and develop “me-better” active molecules, in this study, a series of novel isoxazole-amide derivatives containing an acylhydrazone moiety were synthesized and evaluated for their antiviral activities against tobacco mosaic virus (TMV) and cucumber mosaic virus (CMV). Antiviral bioassays indicated that some of the target compounds exhibited better in vivo antiviral activities against TMV and CMV than those of Ningnanmycin (NNM). Especially, the compound **7t** exhibited the best curative, protection, and inactivation activities against TMV and CMV which were superior to those of NNM. Meanwhile, our present work also revealed that compound **7t** could enhance the defense-related enzyme activity and increase the chlorophyll content in tobacco leaves to induce resistance and enhance plant tolerance to TMV infection.

## 1. Introduction

Tobacco mosaic virus (TMV) and cucumber mosaic virus (CMV) are two significant plant viruses and can cause significant economic losses in various crops, including tobacco, tomato, pepper, cucumbers, and a number of ornamental flowers [1,2]. Ribavirin, Curcumin, and Ningnanmycin (NNM) are successful antiviral agents that are widely used for preventing plant viruses, however, they showed unsatisfactory cure rates (30%–60%) [3]. Therefore, appreciable efficiency for controlling TMV and CMV remain unavailable and development of new effective antiviral agents remains a significant challenge.

**Figure 1:**
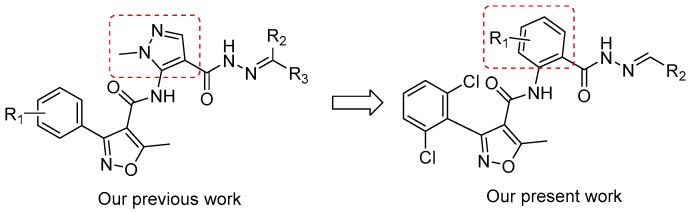
Design route of the target compounds.

In recent years, literature revealed that isoxazole-amide, an important scaffold for synthesis of various active molecules and their derivatives, had a wide range of biological activities, such as antifungal [4], herbicidal [4], antiviral [5], anticancer [6], antibacterial [7], and insecticide [8] activity. Meanwhile, literature revealed that acylhydrazone derivatives had a wide range of biological activities, such as antibacterial [9], fungicidal [10], insecticidal [11,12], and antiviral [13] activity. Specifically, in our previous work, we reported a series of novel isoxazole-amide derivatives containing an acylhydrazone moiety (Figure 1) with potent antiviral activity against TMV [5]. In addition, the phenyl ring especially with electron-withdrawing groups, a privileged structure, represents a key motif in medicinal chemistry because of its ability to improve pharmacological activities including antiviral [14], antifungal [15], antibacterial [16,17], insecticidal [18], and nematicidal [17,19] activity.

As a continuation of our efforts to discover and develop “me-better” active molecules, in this study, we aimed to replace the pyrazole ring with a phenyl ring with electron-withdrawing groups, shown in Figure 1, to build some new isoxazole-amide derivatives containing an acylhydrazone moiety as novel active antiviral agents. Antiviral bioassays indicated that some of the target compounds exhibited better in vivo antiviral activities against TMV and CMV than those of NNM. Especially, the compound **7t** exhibited the best curative, protection, and inactivation activities against TMV and CMV which were superior to those of NNM. Meanwhile, our present work also revealed that compound **7t** could enhance the defense-related enzyme activity and increase the chlorophyll content in tobacco leaves to induce resistance and enhance plant tolerance to TMV infection.

## 2. Results

### 2.1. Chemistry

**Scheme 1:**
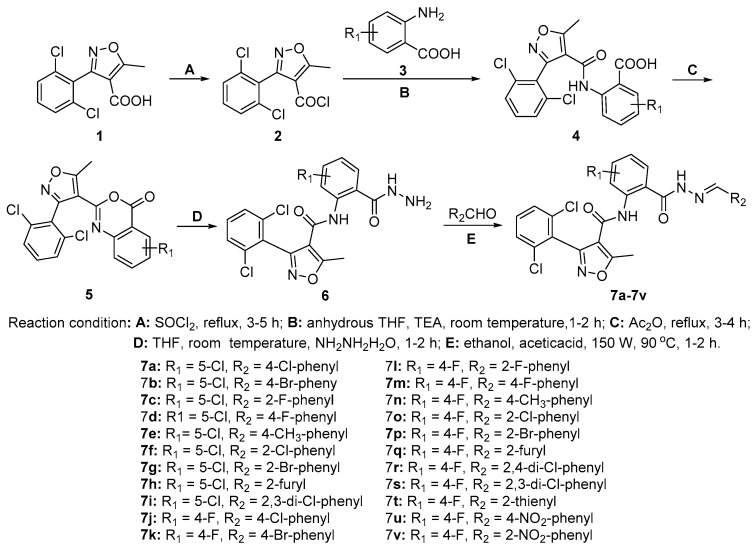
Synthetic route of the target compounds **7a**–**7v**.

Using 3-(2,6-dichlorophenyl)-5-methylisoxazole-4-carboxylic acid as the starting material, as shown in Scheme 1, a series of novel isoxazole-amide derivatives containing an acylhydrazone moiety **7a**–**7v** were synthesized in five steps with yields of 87%−93%. The physical characteristics, IR, ^1^H NMR, ^13^C NMR, ESI-MS, and elemental analysis data for all the target compounds **7a**–**7v** are shown below.

*(E)-N-(5-chloro-2-(2-(4-chlorobenzylidene)hydrazine-1-carbonyl)phenyl)-3-(2,6-dichlorophenyl)-5-methylisoxazole-4-carboxamide* (**7a**). White solid; mp 257−258 °C; yield 90%; ^1^H NMR (500 MHz, DMSO-*d*_6_, ppm) δ: 12.15 (s, 1H, isoxazole–CONH), 11.21 (s, 1H, Ar–CONH), 8.41 (s, 1H, –CH=N–), 8.35 (d, 1H, *J* = 1.50 Hz, Ar–H), 7.82 (dd, 2H, *J*_1_ = *J*_2_ = 8.5 Hz, Ar–H), 7.62–7.49 (m, 6H, Ar–H), 7.37 (dd, 1H, *J*_1_ = 2.50 Hz, *J*_2_ = 2.00 Hz, Ar–H), 2.89 (s, 3H, –CH_3_); ^13^C NMR (125 MHz, DMSO-*d*_6_, ppm) δ: 172.05, 164.19, 162.84, 159.17, 158.45, 148.41, 140.09, 135.46, 135.01, 133.45, 132.89, 130.80, 129.60, 129.47, 128.97, 127.22, 123.87, 121.23, 120.11, 113.80, 13.33; IR (KBr, cm^−1^) *ν*: 3444.87, 3176.76, 2951.09, 1699.29, 1683.86, 1653.00, 1635.64, 1616.35, 1595.13, 1575.84, 1558.48, 1541.12, 1521.84, 1506.41, 1489.08, 1456.26, 1429.25, 1411.89, 1398.39, 1361.74, 1338.60, 1313.52, 1253.73; Anal. Calcd. for C_25_H_16_Cl_4_N_4_O_3_: C 53.41%, H 2.87%, N 9.97%, Found: C 53.47%, H 3.14%, N 10.30%; MS (ESI) *m*/*z*: 563.2 ([M + H]^+^).

*(E)-N-(2-(2-(4-bromobenzylidene)hydrazine-1-carbonyl)-5-chlorophenyl)-3-(2,6-dichlorophenyl)-5-methylisoxazole-4-carboxamide* (**7b**). White solid; mp 259−260 °C; yield 91%; ^1^H NMR (500 MHz, DMSO-*d*_6_, ppm) δ: 12.15 (s, 1H, isoxazole–CONH), 11.19 (s, 1H, Ar–CONH), 8.39 (s, 1H, –CH=N–), 8.35 (d, 1H, *J* = 2.50 Hz, Ar–H), 7.85 (d, 1H, *J* = 8.50 Hz, Ar–H), 7.73–7.68 (m, 4H, Ar–H), 7.61 (d, 2H, *J* = 3.50 Hz, Ar–H), 7.51 (q, 1H, Ar–H), 7.37 (dd, 1H, *J*_1_ = 2.00 Hz, *J*_2_ = 1.50 Hz, Ar–H), 2.89 (s, 3H, –CH_3_); ^13^C NMR (125 MHz, DMSO-*d*_6_, ppm) δ: 172.06, 164.19, 162.85, 159.17, 158.45, 148.50, 140.07, 137.31, 135.01, 133.78, 132.89, 132.51, 130.81, 129.69, 128.97, 127.21, 124.30, 123.88, 121.24, 120.12, 113.80, 13.33; IR (KBr, cm^−1^) *ν*: 3444.87, 3261.63, 3066.82, 1699.29, 1683.86, 1670.35, 1653.00, 1645.28, 1591.27, 1570.06, 1558.48, 1541.12, 1519.91, 1506.41, 1489.08, 1456.26, 1429.25, 1398.39, 1361.74, 1300.02, 1257.29, 1234.44, 1195.87; Anal. Calcd. for C_25_H_16_BrCl_3_N_4_O_3_: C 49.49%, H 2.66%, N 9.23%; Found: C 49.57%, H 3.04%, N 9.54%; MS (ESI) *m*/*z*: 607.7 ([M + H]^+^).

*(E)-N-(5-chloro-2-(2-(2-fluorobenzylidene)hydrazine-1-carbonyl)phenyl)-3-(2,6-dichlorophenyl)-5-methylisoxazole-4-carboxamide* (**7c**). White solid; mp 248−249 °C; yield 91%; ^1^H NMR (500 MHz, DMSO-*d*_6_, ppm) δ: 12.19 (s, 1H, isoxazole–CONH), 11.21 (s, 1H, Ar–CONH), 8.67 (s, 1H, –CH=N–), 8.35 (d, 1H, *J* = 1.50 Hz, Ar–H), 7.98 (t, 1H, *J*_1_ = 6.00 Hz, *J*_2_ = 7.50 Hz, Ar–H), 7.87 (d, 1H, *J* = 8.50 Hz, Ar–H), 7.61 (d, 2H, *J* = 8.00 Hz, Ar–H), 7.56–7.50 (m, 2H, Ar–H), 7.39–7.32 (m, 3H, Ar–H), 2.89 (s, 3H, –CH_3_); ^13^C NMR (125 MHz, DMSO-*d*_6_, ppm) δ: 172.03, 164.15, 160.47, 159.18, 158.46, 142.48, 140.10, 137.37, 135.00, 132.91, 130.80, 128.98, 127.20, 126.93, 125.63, 123.93, 122.09, 121.28, 120.09, 116.74, 116.58, 113.81, 13.32; IR (KBr, cm^−1^) *ν*: 3444.87, 3265.49, 1699.29, 1683.86, 1670.25, 1653.00, 1635.64, 1616.35, 1595.13, 1575.84, 1558.48, 1541.12, 1521.84, 1506.41, 1489.08, 1456.126, 1436.97, 1417.68, 1398.39, 1361.74, 1338.60, 1311.59, 1247.94, 1224.80; Anal. Calcd. for C_25_H_16_Cl_3_FN_4_O_3_: C 55.02%, H 2.95%, N 10.27%, Found: C 55.47%, H 3.22%, N 10.62%; MS (ESI) *m*/*z*: 546.8 ([M + H]^+^).

*(E)-N-(5-chloro-2-(2-(4-fluorobenzylidene)hydrazine-1-carbonyl)phenyl)-3-(2,6-dichlorophenyl)-5-methylisoxazole-4-carboxamide* (**7d**). White solid; mp 245–246 °C; yield 92%; ^1^H NMR (500 MHz, DMSO-*d*_6_, ppm) δ: 12.07 (s, 1H, isoxazole–CONH), 11.21 (s, 1H, Ar–CONH), 8.38 (s, 1H, –CH=N–), 8.32 (d, 1H, *J* = 1.50 Hz, Ar–H), 7.57 (d, 2H, *J* = 8.00 Hz, Ar–H), 7.47 (q, 1H, Ar–H), 7.34–7.28 (m, 3H, Ar–H), 2.85 (s, 3H, –CH_3_); ^13^C NMR (125 MHz, DMSO-*d*_6_, ppm) δ: 172.03, 164.84, 164.15, 162.88, 158.81 (d, *J* = 85.88 Hz), 148.62, 140.09, 137.28, 135.00, 132.88, 131.10, 130.77, 130.11, 130.04, 128.98, 127.23, 123.86, 121.19, 120.05, 116.67, 116.49, 113.80, 13.33; IR (KBr, cm^−1^) *ν*: 3444.87, 3265.49, 1699.29, 1683.86, 1670.25, 1653.00, 1635.64, 1616.35, 1595.13, 1575.84, 1558.48, 1541.12, 1521.84, 1506.41, 1489.08, 1456.26, 1436.97, 1417.68, 1398.39, 1361.74, 1338.60, 1307.74, 12759.29, 1234.44; Anal. Calcd. for C_25_H_16_Cl_3_FN_4_O_3_: C 55.02%, H 2.95%, N 10.27%, Found: C 55.51%, H 3.35%, N 10.37%; MS (ESI) *m*/*z*: 546.8 ([M + H]^+^).

*(E)-N-(5-chloro-2-(2-(4-methylbenzylidene)hydrazine-1-carbonyl)phenyl)-3-(2,6-dichlorophenyl)-5-methylisoxazole-4-carboxamide* (**7e**). White solid; mp 247–248 °C; yield 90%; ^1^H NMR (500 MHz, DMSO-*d*_6_, ppm) δ: 12.05 (s, 1H, isoxazole–CONH), 11.29 (s, 1H, Ar–CONH), 8.39 (s, 1H, –CH=N–), 8.36 (d, 1H, *J* = 2.50 Hz, Ar–H), 7.86 (d, 1H, *J* = 8.50 Hz, Ar–H), 7.69–7.59 (m, 4H, Ar–H), 7.51 (t, 1H, *J*_1_ = 8.00 Hz, *J*_2_ = 8.50 Hz, Ar–H), 7.38–7.30 (m, 3H, Ar–H), 2.90 (s, 3H, –CH_3_), 2.37 (s, 3H, –CH_3_); ^13^C NMR (125 MHz, DMSO-*d*_6_, ppm) δ: 171.97, 164.06, 159.13, 158.52, 149.87, 140.95, 140.10, 137.22, 134.99, 132.88, 131.76, 130.77, 130.09, 128.96, 127.85, 127.25, 123.83, 120.07, 113.82, 21.62, 13.34; IR (KBr, cm^−1^) *ν*: 3444.87, 3265.49, 1699.29, 1683.86, 1670.25, 1653.00, 1635.64, 1616.35, 1595.13, 1575.84, 1558.48, 1541.12, 1521.84, 1506.41, 1489.08, 1473.62, 1456.26, 1436.97, 1429.25, 1398.39, 1375.25, 1338.60, 1317.38, 1253.73, 1234.44; Anal. Calcd. for C_26_H_19_Cl_3_N_4_O_3_: C 57.64%, H 3.53%, N 10.34%, Found: C 57.87%, H 3.78%, N 10.32%; MS (ESI) *m*/*z*: 542.8 ([M + H]^+^).

*(E)-N-(5-chloro-2-(2-(2-chlorobenzylidene)hydrazine-1-carbonyl)phenyl)-3-(2,6-dichlorophenyl)-5-methylisoxazole-4-carboxamide* (**7f**). White solid; mp 241–243 °C; yield 91%; ^1^H NMR (500 MHz, DMSO-*d*_6_, ppm) δ: 12.28 (s, 1H, isoxazole–CONH), 11.21 (s, 1H, Ar–CONH), 8.84 (s, 1H, –CH=N–), 8.35 (d, 1H, *J* = 2.50 Hz, Ar–H), 8.06 (q, 1H, Ar–H), 7.89 (d, 1H, *J* = 8.50 Hz, Ar–H), 7.61 (d, 1H, *J* = 8.00 Hz, Ar–H), 7.57–7.48 (m, 4H, Ar–H), 7.38 (dd, *J*_1_ = *J*_2_ = 1.50 Hz, Ar–H), 2.89 (s, 3H, –CH_3_); ^13^C NMR (125 MHz, DMSO-*d*_6_, ppm) δ: 172.01, 164.23, 159.19, 158.49, 145.65, 140.12, 137.45, 135.01, 133.99, 132.88, 132.43, 131.76, 130.85, 130.56, 128.97, 128.30, 127.51, 127.24, 123.90, 121.30, 120.07, 113.94, 13.32; IR (KBr, cm^−1^) *ν*: 3444.87, 3265.49, 1699.29, 1683.86, 1670.25, 1653.00, 1635.64, 1616.35, 1595.13, 1575.84, 1558.48, 1541.12, 1521.84, 1506.41, 1489.08, 1473.62, 1456.26, 1436.97, 1411.89, 1398.39, 1361.74, 1354.03, 1338.60, 1317.38, 1249.87; Anal. Calcd. for C_25_H_16_Cl_4_N_4_O_3_: C 53.41%, H 2.87%, N 9.97%, Found: C 53.43%, H 3.05%, N 10.22%; MS (ESI) *m*/*z*: 563.2 ([M + H]^+^).

*(E)-N-(2-(2-(2-bromobenzylidene)hydrazine-1-carbonyl)-5-chlorophenyl)-3-(2,6-dichlorophenyl)-5-methylisoxazole-4-carboxamide* (**7g**). White solid; mp 245−247 °C; yield 92%; ^1^H NMR (500 MHz, DMSO-*d*_6_, ppm) δ: 12.32 (s, 1H, isoxazole–CONH), 11.22 (s, 1H, Ar–CONH), 8.80 (s, 1H, –CH=N–), 8.35 (s, 1H, Ar–H), 8.04 (d, 1H, *J* = 7.50 Hz, Ar–H), 7.90 (d, 1H, *J* = 8.00 Hz, Ar–H), 7.74–7.37 (m, 7H, Ar–H), 2.90 (s, 3H, –CH_3_); ^13^C NMR (125 MHz, DMSO-*d*_6_, ppm) δ: 171.98, 164.24, 159.18, 158.50, 147.99, 140.10, 137.40, 135.01, 133.81, 133.25, 132.88, 132.67, 130.87, 128.97, 128.93, 128.77, 127.88, 127.25, 124.38, 123.89, 121.29, 120.09, 113.82, 13.34; IR (KBr, cm^−1^) *ν*: 3446.79, 3307.92, 1699.29, 1683.86, 1670.25, 1653.00, 1635.64, 1616.35, 1595.13, 1575.84, 1558.48, 1541.12, 1521.84, 1506.41, 1489.08, 1456.26, 1436.97, 1429.25, 1411.89, 1398.39, 1361.74, 1338.60, 1313.52, 1276.88, 1247.94, 1234.44; Anal. Calcd. for C_25_H_16_BrCl_3_N_4_O_3_: C 49.49%, H 2.66%, N 9.23%, Found: C 49.88%, H 2.75%, N 9.65%; MS (ESI) *m*/*z*: 607.7([M + H]^+^).

*(E)-N-(5-chloro-2-(2-(furan-2-ylmethylene)hydrazine-1-carbonyl)phenyl)-3-(2,6-dichlorophenyl)-5-methylisoxazole-4-carboxamide* (**7h**). White solid; mp 173−174 °C; yield 89%; ^1^H NMR (500 MHz, DMSO-*d*_6_, ppm) δ: 12.05 (s, 1H, isoxazole–CONH), 11.27 (s, 1H, Ar–CONH), 8.34 (d, 1H, *J* = 2.50 Hz, Ar–H), 8.30 (s, 1H, –CH=N–), 7.91 (d, 1H, *J* = 1.00 Hz, Ar–H), 7.84 (d, 1H, *J* = 9.00 Hz, Ar–H), 7.62 (d, 2H, *J* = 8.00 Hz, Ar–H), 7.53 (q, 1H, Ar–H), 7.37 (dd, 1H, *J*_1_ = *J*_2_ = 2.50 Hz, furyl–H), 7.02 (d, 1H, *J* = 3.50 Hz, furyl–H), 6.68 (q, 1H, furyl–H), 2.90 (s, 3H, –CH_3_); ^13^C NMR (125 MHz, DMSO-**d*_6_*, ppm) *δ*: 171.81, 164.07, 159.11, 158.62, 149.60, 146.23, 140.05, 139.33, 137.26, 134.96, 132.89, 130.71, 128.96, 127.32, 123.88, 121.21, 120.08, 115.17, 113.81, 112.89, 13.32; IR (KBr, cm^−1^) *ν*: 3419.79, 3265.49, 1716.65, 16.95.43, 1683.86, 1662.64, 1653.00, 1635.64, 1616.35, 1595.13, 1575.84, 1558.48, 1541.12, 1521.84, 1506.41, 1489.08, 1473.62, 1456.26, 1436.97, 1429.25, 1398.39, 1361.74, 1338.60, 1313.52, 1249.87, 1233.44; Anal. Calcd. for C_23_H_15_Cl_3_N_4_O_4_: C 53.36%, H 2.92%, N 10.82%, Found: C 53.59%, H 3.25%, N 10.65%; MS (ESI) *m*/*z*: 518.7([M + H]^+^).

*(E)-N-(5-chloro-2-(2-(2,3-dichlorobenzylidene)hydrazine-1-carbonyl)phenyl)-3-(2,6-dichlorophenyl)-5-methylisoxazole-4-carboxamide* (**7i**). White solid; mp 231–232 °C; yield 93%; ^1^H NMR (500 MHz, DMSO-*d*_6_, ppm) δ: 12.36 (s, 1H, isoxazole–CONH), 11.17 (s, 1H, Ar–CONH), 8.86 (s, 1H, –CH=N–), 8.34 (d, 1H, *J* = 1.50 Hz, Ar–H), 8.02 (d, 1H, *J* = 7.00 Hz, Ar–H), 7.88 (d, 1H, *J* = 8.50 Hz, Ar–H), 7.76 (d, 1H, *J* = 8.00 Hz, Ar–H), 7.62 (d, 2H, *J* = 8.00 Hz, Ar–H), 7.54–7.50 (m, 2H, Ar–H), 7.39 (dd, 1H, *J*_1_ = 2.50 Hz, *J*_2_ = 2.00 Hz, Ar–H), 2.89 (s, 3H, –CH_3_); ^13^C NMR (125 MHz, DMSO-*d*_6_, ppm) δ: 172.03, 164.28, 159.20, 158.46, 145.36, 140.08, 137.47, 135.00, 134.22, 132.99, 132.90, 132.47, 130.86, 129.18, 128.98, 127.20, 126.11, 123.95, 113.80, 13.32; IR (KBr, cm^−1^) *ν*: 3446.79, 3265.49, 1716.65, 1699.29, 1683.86, 1670.25, 1653.00, 1635.64, 1616.35, 1595.13, 1575.84, 1558.48, 1541.12, 1521.84, 1506.41, 1489.08, 1473.26, 1456.26, 1436.97, 1419.61, 1398.39, 1361.74, 1350.17, 1313.52, 1288.45, 1246.02, 1226.73; Anal. Calcd. for C_25_H_15_Cl_5_N_4_O_3_: C 50.32%, H 2.53%, N 9.39%, Found: C 50.47%, H 2.95%, N 9.28%; MS (ESI) *m*/*z*: 597.7 ([M + H]^+^).

*(E)-N-(2-(2-(4-chlorobenzylidene)hydrazine-1-carbonyl)-4-fluorophenyl)-3-(2,6-dichlorophenyl)-5-methylisoxazole-4-carboxamide* (**7j**). White solid; mp 258–259 °C; yield 90%; ^1^H NMR (500 MHz, DMSO-*d*_6_, ppm) δ: 12.08 (s, 1H, isoxazole–CONH), 10.77 (s, 1H, Ar–CONH), 8.38 (s, 1H, –CH=N–), 8.14 (q, 1H, Ar–H), 7.96 (s, 1H, Ar–H), 7.79 (d, 2H, *J* = 9.00 Hz, Ar–H), 7.66 (dd, 1H, *J*_1_ = 2.50 Hz, *J*_2_ = 3.50 Hz, Ar–H), 7.58 (q, 4H, Ar–H), 7.51 (q, 1H, Ar–H), 7.45–7.41 (m, 1H, Ar–H), 2.86 (s, 3H, –CH_3_); ^13^C NMR (125 MHz, DMSO-*d*_6_, ppm) δ: 171.54, 163.56, 162.84, 159.00 (d, *J* = 15.50 Hz), 158.47, 157.13, 148.26, 135.44, 135.01, 134.67, 133.45, 132.80, 129.59, 129.47, 128.93, 127.35, 124.73, 119.47, 119.29, 115.89, 115.70, 113.94, 13.25; IR (KBr, cm^−1^) *ν*: 3444.87, 3248.13, 1683.86, 1653.00, 1616.35, 1595.13, 1558.48, 1521.84, 1448.54, 1417.68, 1404.18, 1386.82, 1328.95, 1267.23, 1244.09, 1213.23. Anal. Calcd. for C_25_H_16_FCl_3_N_4_O_3_: C 55.02%, H 2.95%, N 10.27%, Found: C 55.36%, H 3.06%, N 10.43%; MS (ESI) *m*/*z*: 546.8 ([M + H]^+^).

*(E)-N-(2-(2-(4-bromobenzylidene)hydrazine-1-carbonyl)-4-fluorophenyl)-3-(2,6-dichlorophenyl)-5-methylisoxazole-4-carboxamide* (**7k**). White solid; mp 265–267 °C; yield 90%; ^1^H NMR (500 MHz, DMSO-*d*_6_, ppm) δ: 12.08 (s, 1H, isoxazole–CONH), 10.75 (s, 1H, Ar–CONH), 8.36 (s, 1H, –CH=N–), 8.13 (q, 1H, Ar–H), 7.73–7.69 (m, 4H, Ar–H), 7.59 (d, 2H, *J* = 8.00 Hz, Ar–H), 7.50 (q, 1H, Ar–H), 7.44–7.40 (m, 1H, Ar–H), 2.86 (s, 3H, –CH_3_); ^13^C NMR (125 MHz, DMSO-*d*_6_, ppm) δ: 171.64, 163.69, 162.84, 159.04, 158.71 (d, *J* = 58.38 Hz), 157.95, 157.13, 148.34, 135.00, 134.65, 133.78, 132.80, 132.50, 132.48, 129.68, 129.66, 128.93, 128.91, 127.35, 124.73, 124.27, 119.47, 113.94, 13.19; IR (KBr, cm^−1^) *ν*: 3444.87, 3248.13, 1683.86, 1670.35, 1653.00, 1635.64, 1589.34, 1558.48, 1521.84, 1458.18, 1417.68, 1361.74, 1319.31, 1271.09, 1244.09, 1201.65; Anal. Calcd. for C_25_H_16_BrCl_2_FN_4_O_3_: C 50.87%, H 2.73%, N 9.49%, Found: C 51.08%, H 2.96%, N 9.78%; MS (ESI) *m*/*z*: 591.2 ([M + H]^+^).

*(E)-3-(2,6-dichlorophenyl)-N-(4-fluoro-2-(2-(2-fluorobenzylidene)hydrazine-1-carbonyl)phenyl)-5-methylisoxazole-4-carboxamide* (**7l**). White solid; mp 248−249 °C; yield 89%; ^1^H NMR (500 MHz, DMSO-*d*_6_, ppm) δ: 12.11 (s, 1H, isoxazole–CONH), 10.78 (s, 1H, Ar–CONH), 8.65 (s, 1H, –CH=N–), 8.16 (q, 1H, Ar–H), 7.98 (s, 1H, Ar–H), 7.69 (d, 1H, *J* = 7.00 Hz, Ar–H), 7.60 (d, 1H, *J* = 7.00 Hz, Ar–H), 7.52 (q, 1H, Ar–H), 7.42 (d, 1H, *J* = 9.00 Hz, Ar–H), 7.33 (t, 1H, *J*_1_ = 8.00 Hz, *J*_2_ = 9.00 Hz, Ar–H), 2.87 (s, 3H, –CH_3_); ^13^C NMR (125 MHz, DMSO-*d*_6_, ppm) δ: 171.64, 163.52, 158.72 (d, *J* = 61.63 Hz), 157.17 (d, *J* = 56.00 Hz), 142.35, 135.02, 134.71, 133.00, 132.93, 132.79, 128.93, 128.59, 128.49, 127.35, 126.94, 125.60, 124.82, 124.63, 122.02, 119.36, 116.55, 115.69, 113.96, 13.24; IR (KBr, cm^−1^) *ν*: 3444.87, 3244.27, 1683.86, 1670.35, 1653.00, 1635.64, 1616.35, 1589.34, 1558.48, 1521.84, 1458.18, 1436.97, 1411.89, 1361.74, 1319.31, 1305.81, 1238.30, 1228.66; Anal. Calcd. for C_25_H_16_F_2_Cl_2_N_4_O_3_: C 56.73%, H 3.05%, N 10.58%, Found: C 57.05%, H 3.14%, N 10.91%; MS (ESI) *m*/*z*: 530.3 ([M + H]^+^).

*(E)-3-(2,6-dichlorophenyl)-N-(4-fluoro-2-(2-(4-fluorobenzylidene)hydrazine-1-carbonyl)phenyl)-5-methylisoxazole-4-carboxamide* (**7m**). White solid; mp 234−235 °C; yield 91%; ^1^H NMR (500 MHz, DMSO-*d*_6_, ppm) δ: 12.03 (s, 1H, isoxazole–CONH), 10.80 (s, 1H, Ar–CONH), 8.39 (s, 1H, –CH=N–), 8.15 (q, 1H, Ar–H), 7.83 (q, 2H, Ar–H), 7.71 (dd, 1H, *J*_1_ = *J*_2_ = 3.00 Hz, Ar–H), 7.60 (d, 2H, *J* = 3.00 Hz, Ar–H), 7.50 (q, 1H, Ar–H), 7.44–7.40 (m, 1H, Ar–H), 7.34 (t, 2H, *J*_1_ = 8.50 Hz, *J*_2_ = 9.50 Hz, Ar–H), 2.87 (s, 3H, –CH_3_); ^13^C NMR (125 MHz, DMSO-*d*_6_, ppm) δ: 171.64, 164.86, 162.20 (d, *J* = 79.88 Hz), 158.69 (d, *J* = 56.00 Hz), 157.11, 148.50, 135.01, 134.70, 132.80, 131.90, 130.10, 130.03, 128.93, 127.35, 124.73, 124.66, 119.43, 119.26, 116.66, 116.49, 115.87, 113.94, 13.25; IR (KBr, cm^−1^) *ν*: 3444.87, 3244.27, 1683.86, 1670.35, 1653.00, 1635.64, 1602.85, 1558.48, 1541.12, 1521.84, 1506.41, 1458.18, 1436.97, 1361.74, 1317.38, 1273.02, 1238.30, 1211.30; Anal. Calcd. for C_25_H_16_F_2_Cl_2_N_4_O_3_: C 56.73%, H 3.05%, N 10.58%, Found: C 56.68%, H 3.32%, N 10.69%; MS (ESI) *m*/*z*: 530.3 ([M + H]^+^).

*(E)-3-(2,6-dichlorophenyl)-N-(4-fluoro-2-(2-(4-methylbenzylidene)hydrazine-1-carbonyl)phenyl)-5-methylisoxazole-4-carboxamide* (**7n**). White solid; mp 267–269 °C; yield 87%; ^1^H NMR (500 MHz, DMSO-*d*_6_, ppm) δ: 11.96 (s, 1H, isoxazole–CONH), 10.82 (s, 1H, Ar–CONH), 8.36 (s, 1H, –CH=N–), 8.16 (q, 1H, Ar–H), 7.66 (t, 3H, *J*_1_ = 6.00 Hz, *J*_2_ = 8.00 Hz, Ar–H), 7.58 (t, 1H, *J*_1_ = 9.00 Hz, *J*_2_ = 8.50 Hz, Ar–H), 7.44–7.40 (m, 1H, Ar–H), 7.31 (d, 1H, *J* = 8.00 Hz, Ar–H), 2.87 (s, 3H, –CH_3_), 2.37 (s, 3H, –CH_3_); ^13^C NMR (125 MHz, DMSO-*d*_6_, ppm) δ: 171.60, 163.44, 158.70 (d, *J* = 48.88 Hz), 157.10, 149.77, 140.92, 135.00, 134.74, 132.78, 131.78, 130.09, 128.92, 127.83, 127.81, 127.38, 124.55, 119.38, 119.21, 115.85, 115.65, 113.95, 21.61, 13.26; IR (KBr, cm^−1^) *ν*: 3444.87, 3246.20, 1683.86, 1668.43, 1637.56, 1610.56, 1558.48, 1521.84, 1508.33, 1458.18, 1429.25, 1413.82, 1363.67, 1313.52, 1274.95, 1238.30, 1199.72; Anal. Calc. for C_26_H_19_FCl_2_N_4_O_3_: C 59.44%, H 3.65%, N 10.66%, Found: C 59.58%, H 3.55%, N 10.85%; MS (ESI) *m*/*z*: 526.4 ([M + H]^+^).

*(E)-N-(2-(2-(2-chlorobenzylidene)hydrazine-1-carbonyl)-4-fluorophenyl)-3-(2,6-dichlorophenyl)-5-methylisoxazole-4-carboxamide* (**7o**). White solid; mp 250–251 °C; yield 88%; ^1^H NMR (500 MHz, DMSO-*d*_6_, ppm) δ: 12.20 (s, 1H, isoxazole–CONH), 10.77 (s, 1H, Ar–CONH), 8.81 (s, 1H, –CH=N–), 8.12 (d, 1H, *J* = 5.00 Hz, Ar–H), 8.04 (s, 1H, Ar–H), 7.70 (d, 1H, *J* = 6.50 Hz, Ar–H), 7.60–7.44 (m, 7H, Ar–H), 2.87 (s, 3H, –CH_3_); ^13^C NMR (125 MHz, DMSO-*d*_6_, ppm) δ: 171.59, 163.58, 158.73 (d, *J* = 59.63 Hz), 145.51, 135.01, 134.68, 133.96, 132.79, 132.42, 131.75, 130.57, 128.93, 128.82, 128.29, 127.50, 127.36, 124.91, 124.73, 119.55, 119.38, 115.92, 115.72, 113.97, 13.24; IR (KBr, cm^−1^) *ν*: 3444.87, 3244.27, 1683.86, 1653.00, 1635.64, 1616.35, 1558.48, 1541.12, 1521.84, 1506.41, 1456.26, 1429.25, 1398.39, 1361.74, 1319.31, 1269.16, 1236.37, 1197.79; Anal. Calcd. for C_25_H_16_FCl_3_N_4_O_3_: C 55.02%, H 2.95%, N 10.27%, Found: C 55.14%, H 3.15%, N 10.36%; MS (ESI) *m*/*z:* 546.8 ([M + H]^+^).

*(E)-N-(2-(2-(2-bromobenzylidene)hydrazine-1-carbonyl)-4-fluorophenyl)-3-(2,6-dichlorophenyl)-5-methylisoxazole-4-carboxamide* (**7p**). White solid; mp 249–250 °C; yield 89%; ^1^H NMR (500 MHz, DMSO-*d*_6_, ppm) δ: 12.23 (s, 1H, isoxazole–CONH), 10.77 (s, 1H, Ar–CONH), 8.77 (s, 1H, –CH=N–), 8.13 (q, 1H, Ar–H), 8.03 (dd, 1H, *J*_1_ = 1.00 Hz, *J*_2_ = 1.50 Hz, Ar–H), 7.74–7.69 (m, 2H, Ar–H), 7.59 (d, 2H, *J* = 8.00 Hz, Ar–H), 7.53–7.49 (m, 2H, Ar–H), 7.44–7.39 (m, 2H, Ar–H), 2.86 (s, 3H, –CH_3_); ^13^C NMR (125 MHz, DMSO-*d*_6_, ppm) δ: 171.57, 163.60, 159.11, 158.96, 158.51, 158.74 (d, *J* = 57.25 Hz), 147.83, 135.01, 134.67, 133.81, 133.25, 132.79, 132.66, 128.93, 128.77, 127.88, 127.37, 124.92, 124.92, 124.71, 124.35, 119.54, 119.36, 115.93, 115.74, 113.97, 13.26; IR (KBr, cm^−1^) *ν*: 3444.87, 3224.98, 3066.82, 1683.86, 1653.00, 1635.64, 1608.63, 1591.27, 1558.48, 1541.12, 1521.84, 1506.41, 1458.18, 1429.25, 1417.68, 1363.67, 1321.24, 1280.73, 1244.09, 1207.44; Anal. Calcd. for C_25_H_16_BrCl_2_FN_4_O_3_: C 50.87%, H 2.73%, N 9.49%, Found: C 51.03%, H 2.74%, N 9.76%; MS (ESI) *m*/*z*: 591.2 ([M + H]^+^).

*(E)-3-(2,6-dichlorophenyl)-N-(4-fluoro-2-(2-(furan-2-ylmethylene)hydrazine-1-carbonyl)phenyl)-5-methylisoxazole-4-carboxamide* (**7q**). Brown solid; mp 232–234 °C; yield 87%; ^1^H NMR (500 MHz, DMSO-*d*_6_, ppm) δ: 11.92 (s, 1H, isoxazole–CONH), 10.77 (s, 1H, Ar–CONH), 8.23 (s, 1H, –CH=N–), 8.10 (q, 1H, Ar–H), 7.86 (d, 1H, *J* = 1.50 Hz, Ar–H), 7.61 (dd, 1H, *J*_1_ = 2.50 Hz, *J*_2_ = 3.00 Hz, Ar–H), 7.56 (d, 2H, *J* = 8.00 Hz, furyl–H), 7.47 (q, 1H, furyl–H), 7.40–7.36 (m, 1H, Ar–H), 2.83 (s, 3H, –CH_3_); ^13^C NMR (125 MHz, DMSO-*d*_6_, ppm) δ: 171.47, 163.44, 158.74 (d, *J* = 37.00 Hz), 157.12, 149.63, 146.19, 139.25, 134.98, 134.70, 132.79, 128.91, 127.44, 124.82, 124.75, 124.64, 119.43, 119.26, 115.78, 115.59, 115.08, 113.95, 112.87, 13.24; IR (KBr, cm^−1^) *ν*: 3444.87, 3224.98, 3066.82, 1683.86, 1653.00, 1635.64, 1608.63, 1595.13, 1575.84, 1558.48, 1541.12, 1521.84, 1506.41, 1458.18, 1429.25, 1409.95, 1394.53, 1338.60, 1319.31, 1271.09, 1242.16, 1199.72; Anal. Calcd. for C_23_H_15_FCl_2_N_4_O_4_: C 55.11%, H 3.02%, N 11.18%, Found: C 55.43%, H 3.15%, N 11.57%; MS (ESI) *m*/*z*: 502.3 ([M + H]^+^).

*(E)-N-(2-(2-(2,4-dichlorobenzylidene)hydrazine-1-carbonyl)-4-fluorophenyl)-3-(2,6-dichlorophenyl)-5-methylisoxazole-4-carboxamide* (**7r**). White solid; mp 231–233 °C; yield 92%; ^1^H NMR (500 MHz, DMSO-*d*_6_, ppm) δ: 12.23 (s, 1H, isoxazole–CONH), 10.75 (s, 1H, Ar–CONH), 8.75 (s, 1H, –CH=N–), 8.13 (q, 1H, Ar–H), 8.05 (d, 1H, *J* = 8.50 Hz, Ar–H), 7.75 (d, 1H, *J* = 2.00 Hz, Ar–H), 7.69 (dd, 1H, *J*_1_ = *J*_2_ = 3.00 Hz, Ar–H), 7.64 (q, 2H, Ar–H), 7.50 (q, 2H, Ar–H), 7.45–7.42 (m, 1H, Ar–H), 2.86 (s, 3H, –CH_3_); ^13^C NMR (125 MHz, DMSO-*d*_6_, ppm) δ: 171.62, 163.59, 158.73 (d, *J* = 64.38 Hz), 144.42, 136.02, 135.02, 134.69, 134.65, 132.78, 130.89, 130.03, 128.92, 128.69, 127.36, 124.95, 124.89, 124.89, 124.68, 119.61, 119.43, 115.88, 115.69, 113.95, 13.24; IR (KBr, cm^−1^) *ν*: 3444.87, 3089.11, 1683.86, 1674.21, 1653.00, 1635.64, 1616.35, 1575.84, 1558.48, 1541.12, 1521.84, 1506.41, 1473.62, 1458.18, 1436.97, 1416.68, 1361.74, 1338.60, 1319.31, 1271.09, 1240.23, 1211.30; Anal. Calcd. for C_25_H_15_Cl_4_FN_4_O_3_: C 51.75%, H 2.61%, N 9.66%, Found: C 51.83%, H 3.03%, N 9.78%; MS (ESI) *m*/*z*: 581.2 ([M + H]^+^).

*(E)-N-(2-(2-(2,3-dichlorobenzylidene)hydrazine-1-carbonyl)-4-fluorophenyl)-3-(2,6-dichlorophenyl)-5-methylisoxazole-4-carboxamide* (**7s**). White solid; mp 176–177 °C; yield 90%; ^1^H NMR (500 MHz, DMSO-*d*_6_, ppm) δ: 12.27 (s, 1H, isoxazole–CONH), 10.73 (s, 1H, Ar–CONH), 8.82 (s, 1H, –CH=N–), 8.11 (q, 1H, Ar–H), 8.01 (dd, 1H, *J*_1_ = *J*_2_ = 1.50 Hz, Ar–H), 7.76 (dd, 1H, *J*_1_ = *J*_2_ = 1.00 Hz, Ar–H), 7.69 (dd, 1H, *J*_1_ = 2.50 Hz, *J*_2_ = 3.00 Hz, Ar–H), 7.60 (d, 2H, *J* = 7.50 Hz, Ar–H), 7.52–7.49 (m, 2H, Ar–H), 7.44 (t, 1H, *J*_1_ = 6.00 Hz, *J*_2_ = 8.50 Hz, Ar–H), 2.86 (s, 3H, –CH_3_); ^13^C NMR (125 MHz, DMSO-*d*_6_, ppm) δ: 171.61, 163.62, 158.74 (d, *J* = 66.76 Hz), 157.13, 145.21, 135.00, 134.62, 134.23, 132.99, 132.81, 132.46, 131.76, 129.18, 128.94, 128.82, 127.34, 126.11, 124.90, 124.78, 119.44, 115.90, 115.67, 113.96, 13.24; IR (KBr, cm^−1^) *ν*: 3444.87, 1734.01, 1716.05, 1683.86, 1674.21, 1653.00, 1635.64, 1616.35, 1575.84, 1558.48, 1541.12, 1521.84, 1506.41, 1489.05, 1473.62, 1458.18, 1436.97, 1429.25, 1416.61, 1361.74, 1338.60, 1319.31, 1271.09, 1226.21; Anal. Calcd. for C_25_H_15_Cl_4_FN_4_O_3_: C 51.75%, H 2.61%, N 9.66%, Found: C 51.93%, H 2.87%, N 9.70%; MS (ESI) *m*/*z*: 581.2 ([M + H]^+^).

*(E)-3-(2,6-dichlorophenyl)-N-(4-fluoro-2-(2-(thiophen-2-ylmethylene)hydrazine-1-carbonyl)phenyl)-5-methylisoxazole-4-carboxamide* (**7t**). Brown solid; mp 246–248 °C; yield 87%; ^1^H NMR (500 MHz, DMSO-*d*_6_, ppm) δ: 11.96 (s, 1H, isoxazole–CONH), 10.76 (s, 1H, Ar–CONH), 8.58 (s, 1H, –CH=N–), 8.14 (q, 1H, Ar–H), 7.74 (d, 1H, *J* = 4.50 Hz, Ar–H), 7.64 (dd, 1H, *J*_1_ = *J*_2_ = 3.00 Hz, Ar–H), 7.60 (d, 1H, *J* = 1.50 Hz, Ar–H), 7.59 (s, 1H, thienyl–H), 7.54–7.49 (m, 2H, Ar–H), 7.44–7.40 (m, 1H, thienyl–H), 7.18 (q, 1H, thienyl–H), 2.86 (s, 3H, –CH_3_); ^13^C NMR (125 MHz, DMSO-*d*_6_, ppm) δ: 171.70, 163.28, 158.98 (d, *J* = 63.25 Hz), 157.10, 144.65, 139.19, 135.01, 134.67, 132.81, 132.25, 130.11, 128.94, 128.55, 128.41, 127.32, 124.75, 124.61, 119.39, 119.22, 115.82, 115.63, 113.95, 13.27; IR (KBr, cm^−1^) *ν*: 3444.87, 3186.40, 3047.53, 1683.86, 1674.21, 1653.00, 1635.64, 1608.13, 1589.34, 1558.48, 1541.12, 1521.84, 1506.41, 1489.05, 1473.62, 1458.18, 1436.97, 1409.96, 1373.32, 1319.31, 1271.09, 1238.30; Anal. Calcd. for C_23_H_15_Cl_2_FN_4_O_3_S: C 53.40, H 2.92, N 10.83, Found: C 53.62%, H 3.05%, N 11.18%; MS (ESI) *m*/*z*: 518.4 ([M + H]^+^).

*(E)-3-(2,6-dichlorophenyl)-N-(4-fluoro-2-(2-(4-nitrobenzylidene)hydrazine-1-carbonyl)phenyl)-5-methylisoxazole-4-carboxamide* (**7u**). Light yellow solid; mp 269–271 °C; yield 89%; ^1^H NMR (500 MHz, DMSO-*d*_6_, ppm) δ: 12.29 (s, 1H, isoxazole–CONH), 10.70 (s, 1H, Ar–CONH), 8.48 (s, 1H, –CH=N–), 8.34 (d, 1H, *J* = 7.50 Hz, Ar–H), 8.12 (q, 1H, Ar–H), 8.03 (d, 2H, *J* = 7.50 Hz, Ar–H), 7.96 (s, 1H, Ar–H), 7.67 (dd, 1H, *J*_1_ = 2.50 Hz, *J*_2_ = 3.00 Hz, Ar–H), 7.60 (d, 2H, *J* = 8.00 Hz, Ar–H), 7.52–7.42 (m, 1H, Ar–H), 2.74 (s, 3H, –CH_3_); ^13^C NMR (125 MHz, DMSO-*d*_6_, ppm) δ: 171.68, 163.77, 162.83, 158.73 (d, *J* = 76.30 Hz), 158.42, 157.21, 148.61, 146.93, 140.76, 135.01, 134.57, 132.80, 128.94, 128.76, 127.33, 125.03, 124.68, 124.45, 119.59, 119.42, 115.98, 115.78, 113.95, 13.25; IR (KBr, cm^−1^) *ν*: 3481.51, 3265.49, 3186.40, 3047.53, 1683.86, 1674.21, 1653.00, 1635.64, 1616.35, 1595.31, 1558.48, 1541.12, 1521.84, 1506.41, 1489.05, 1458.18, 1436.97, 1417.68, 1361.74, 1342.46, 1317.38, 1303.88, 1271.09, 1242.16, 1213.21, 1203.58; Anal. Calcd. for C_25_H_16_FCl_2_N_5_O_5_: C 53.97%, H 2.90%, N 12.59%, Found: C 54.13%, H 3.04%, N 12.81%; MS (ESI) *m*/*z*: 557.3 ([M + H]^+^).

*(E)-3-(2,6-dichlorophenyl)-N-(4-fluoro-2-(2-(2-nitrobenzylidene)hydrazine-1-carbonyl)phenyl)-5-methylisoxazole-4-carboxamide* (**7v**). Light yellow solid; mp 266–268 °C; yield 89%; ^1^H NMR (500 MHz, DMSO-*d*_6_, ppm) δ: 12.26 (s, 1H, isoxazole–CONH), 10.71 (s, 1H, Ar–CONH), 8.59 (s, 1H, –CH=N–), 8.49 (d, 1H, *J* = 6.00 Hz, Ar–H), 8.30 (s, 1H, Ar–H), 8.15 (q, 2H, Ar–H), 8.12 (q, 1H, Ar–H), 7.78 (q, 1H, Ar–H), 7.67–7.44 (m, 5H, Ar–H), 2.85 (s, 3H, –CH_3_); ^13^C NMR (125 MHz, DMSO-*d*_6_, ppm) δ: 171.71, 163.74, 162.84, 158.71 (d, *J* = 77.50 Hz), 157.20, 148.82, 147.00, 136.38, 135.02, 134.57, 134.08, 132.81, 131.11, 128.93, 127.33, 125.13, 124.99, 121.69, 119.54, 119.37, 115.97, 115.78, 113.95, 13.27; IR (KBr, cm^−1^) *ν*: 3444.87, 3265.49, 3066.82, 1683.86, 1674.21, 1653.00, 1635.64, 1616.35, 1595.31, 1575.84, 1558.48, 1541.12, 1521.84, 1506.41, 1489.05, 1473.62, 1456.26, 1436.97, 1417.68, 1398.39, 1350.17, 1317.38, 1271.09, 1238.30, 1201.68; Anal. Calcd. for C_25_H_16_FCl_2_N_5_O_5_: C 53.97%, H 2.90%, N 12.59%, Found: C 54.05%, H 3.23%, N 12.71%; MS (ESI) *m*/*z*: 557.3 ([M + H]^+^).

### 2.2. Biological Evaluations

**Figure 2:**
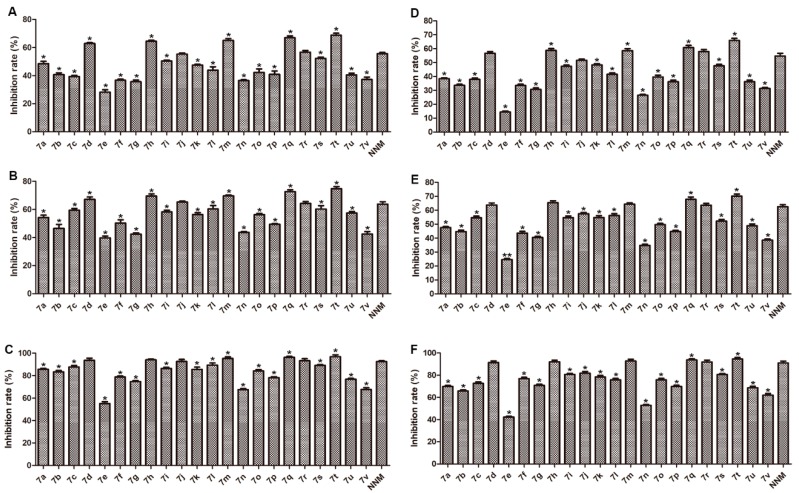
The preliminary in vivo antiviral activities against tobacco mosaic virus (TMV) and cucumber mosaic virus (CMV) of the target compounds **7a**–**7v** at 500 mg/L. (**A**) Curative activity against TMV, (**B**) Protection activity against TMV, (**C**) Inactivation activity against TMV, (**D**) Curative activity against CMV, (**E**) Protection activity against CMV, (**F**) Inactivation activity against CMV. * *p* < 0.05, ** *p* < 0.01. Vertical bars refer to mean ± SD (*n* = 3).

In this study, the curative, protection, and inactivation activities of the target compounds **7a**–**7v** against TMV and CMV in vivo were performed according to the previously reported literature references [20,21]. Among the target compounds evaluated, as shown in Figure 2 and Table S1, compounds **7d**, **7h**, **7m**, **7q**, and **7t** exhibited good curative activity against TMV, with inhibition rates of 62.9%, 64.5%, 65.1%, 67.0%, and 68.8%, respectively, which were even better than that of NNM (55.6%); compounds **7d**, **7h**, **7m**, **7q**, and **7t** exhibited significantly greater protection activity against TMV, with inhibition rates of 67.2%, 69.6%, 69.7%, 72.7%, and 74.8%, respectively, compared with NNM (63.8%); compounds **7m**, **7q**, and **7t** showed superior inactivation activity, with inhibition rates of 95.3%, 96.3%, and 96.9%, respectively, compared with NNM (92.5%). Meanwhile, compounds **7h**, **7m**, **7q**, and **7t** showed remarkable curative activity against CMV, with inhibition rates of 58.8%, 58.6%, 60.8%, and 65.9%, respectively, which were even better than that of NNM (54.7%); compounds **7q** and **7t** showed remarkable protection activity against CMV, with inhibition rates of 67.8% and 70.2%, respectively, which were better than that of NNM (62.6%); compounds **7q** and **7t** showed remarkable inactivation activity against CMV, with inhibition rates of 93.8% and 94.6%, respectively, which were better than that of NNM (91.0%). Preliminary bioassay results showed that compound **7t** revealed the best curative, protection, and inactivation activities against TMV and CMV at 500 mg/L (Figures S1 and S2) compared with those of NNM and the other target compounds.

**Figure 3:**
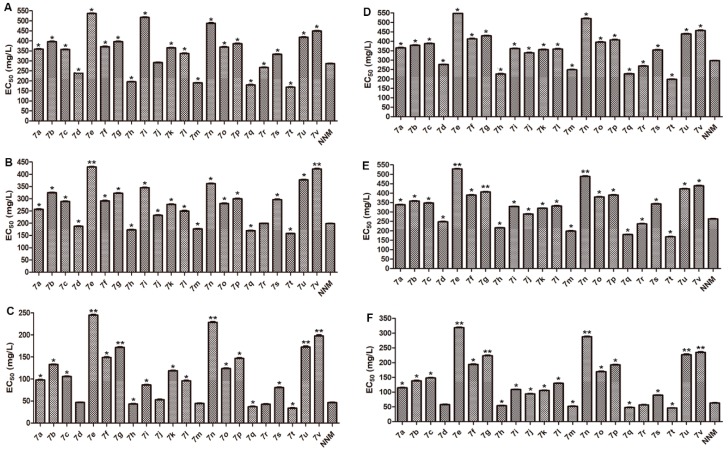
The EC_50_ values of the target compounds **7a–7v** against TMV and CMV in vivo. (**A**) Curative activity against TMV, (**B**) Protection activity against TMV, (**C**) Inactivation activity against TMV, (**D**) Curative activity against CMV, (**E**) Protection activity against CMV, (**F**) Inactivation activity against CMV. * *p* < 0.05, ** *p* < 0.01. Vertical bars refer to mean ± SD (*n* = 3).

Based on the preliminary bioassays, the 50% effective concentration (EC_50_) values of curative, protection, and inactivation activities against TMV and CMV of the target compounds were tested and are listed in Figure 3 and Tables S2 and S3. Bioassay results showed that some of the target compounds showed moderate to good antiviral activities against TMV and CMV. Especially, Figure 3 and Table S2 showed that compounds **7d**, **7h**, **7m**, **7q**, **7r**, and **7t** exhibited excellent curative activity against TMV, with the EC_50_ values of 238.9, 195.5, 189.9, 179.6, 266.8, and 168.5 mg/L, respectively, which were even better that of NNM (286.4 mg/L); compounds **7d**, **7h**, **7m**, **7q**, and **7t** revealed better protection activity against TMV, with the EC_50_ values of 187.2, 172.6, 176.5, 169.2, and 157.6 mg/L, than that of NNM (198.2 mg/L); compounds **7h**, **7q**, and **7t** demonstrated remarkable inactivation activity against TMV, with the EC_50_ values of 43.1, 36.9, and 33.7 mg/L, which were superior to that of NNM (46.3 mg/L). Meanwhile, Figure 3 and Table S3 also showed that compounds **7d**, **7h**, **7m**, **7q**, **7r**, and **7t** exhibited excellent curative activity against CMV, with the EC_50_ values of 276.5, 226.4, 248.5, 227.3, 268.5, and 197.9 mg/L, respectively, which were even better that of NNM (297.3 mg/L); compounds **7d**, **7h**, **7m**, **7q**, **7r**, and **7t** revealed better protection activity against CMV, with the EC_50_ values of 247.8, 215.8, 198.3, 179.7, 236.9, and 168.4 mg/L, than that of NNM (263.4 mg/L); compounds **7h**, **7m**, **7q**, and **7t** demonstrated remarkable inactivation activity against CMV, with the EC_50_ values of 53.7, 51.8, 47.6, and 45.8 mg/L, which were superior to that of NNM (62.8 mg/L).

### 2.3. Effect on Chlorophyll Contents

**Figure 4:**
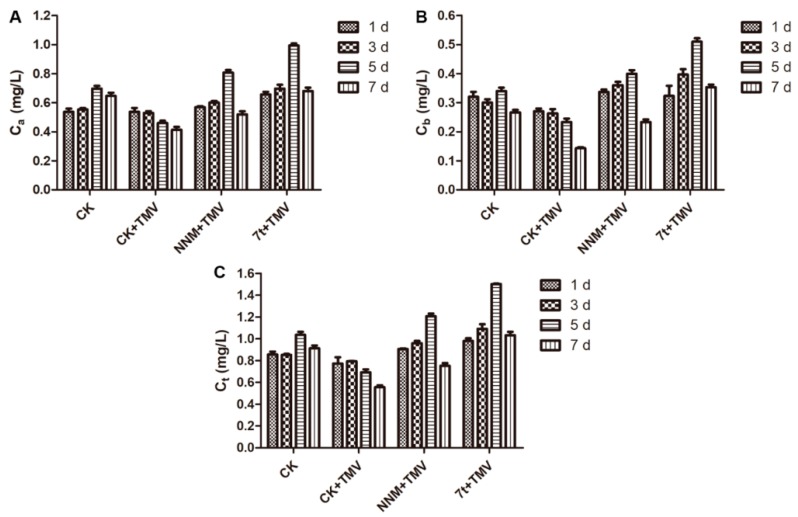
Effects of compound **7t** on chlorophyll a (C_a_) (**A**), chlorophyll b (C_b_) (**B**), and total chlorophyll content (C_t_) (**C**) contents in tobacco leaves. Vertical bars refer to mean ± SD (*n* = 3). CK, negative control.

Figure 4 showed that the chlorophylls included chlorophyll a (C_a_) and chlorophyll b (C_b_), and total chlorophyll content (C_t_) contents of tobacco plant decreased gradually with TMV inoculation from day 1 to day 5. However, in TMV-inoculated tobacco leaves treated with compound **7t**, chlorophyll contents increased from day 1 to day 5 and reached the highest value at day 5. Meanwhile, it is a remarkable fact that variation in trends of **7t** + TMV treatment surpassed that of NNM + TMV treatment.

### 2.4. Effect on Defensive Enzyme Activities

**Figure 5:**
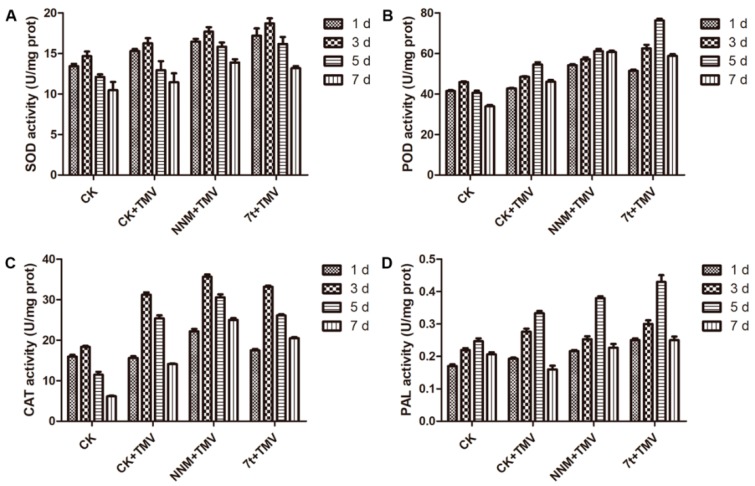
Effects of compound **7t** on superoxide dismutase (SOD) (**A**), peroxidase (POD) (**B**), catalase (CAT) (**C**), and phenylalanine ammonia lyase (PAL) (**D**) activity in tobacco leaves. Vertical bars refer to mean ± SD (*n* = 3). CK, negative control.

Figure 5A shows that superoxide dismutase (SOD) activity of **7t** + TMV treatment group, which was higher than those of CK, CK + TMV, and NNM + TMV groups, increased at day 1 to day 3 and reached the highest value at day 3. Figure 5B shows that peroxidase (POD) activity of **7t** + TMV treatment group, which was higher than those of CK, CK + TMV, and NNM + TMV groups, increased at day 1 to day 5 and reached the highest value at day 5. Meanwhile, Figure 5C shows that catalase (CAT) activity of **7t** + TMV treatment group, which was higher than those of CK and CK + TMV groups but lower than that of NNM + TMV group, increased at day 1 to day 3 and reached the highest value at day 3. In addition, Figure 5D shows that phenylalanine ammonia lyase (PAL) activity of **7t** + TMV treatment group, which was higher than those of CK, CK + TMV, and NNM + TMV groups, increased at day 1 to day 5 and reached the highest value at day 5.

## 3. Discussion

### 3.1. Structure–Activity Relationship (SAR) Analysis

As an extension of this approach to discover and develop “me-better” active molecules, in this study, the SAR were analyzed based on the antiviral activities of the target compounds against TMV and CMV. SAR analysis showed that the type and position of the substituent group R_1_ at the phenyl ring and the type of the substituent group R_2_ had an important effect on the antiviral activities of the target compounds. First, compared with the same substituent on the R_2_ substituent group, the antiviral activities against TMV and CMV of the corresponding compounds with the –F substituent group at 4-position are higher than the –Cl substituent group at 5-position in the order of **7j** > **7a**, **7k** > **7b**, **7g** > **7h**, and **7s** > **7i**. Second, compared with the same substituent on the R_1_ substituent group, the smaller electron-withdrawing groups at the 2- or 4-position of phenyl are beneficial to the improvement of antiviral activities against TMV and CMV in the order of **7d** > **7a** > **7b**, **7c** > **7f** > **7g**, **7m** > **7j** > **7k**, and **7l** > **7o** > **7p**. Third, compared with the same substituent on the R_1_ and R_2_ substituent groups, the antiviral activities against TMV and CMV of the corresponding compounds with the R_2_ substituent group at 4-position of phenyl are higher than those at 2-position in the order of **7d** > **7c** and **7m** > **7l**. Meanwhile, when replacing the pyrazole ring with the phenyl ring, the corresponding compounds synthesized in this work exhibited excellent bioactivities against TMV compared with the bioassay results of the compounds that were reported in our previous work [5].

### 3.2. Physiological and Biochemical Responses of Tobacco

Plants face infection by various aggressive pathogens over their lifetime. To protect themselves during pathogenic infections, plants have evolved unique immune systems and developed various defense responses; such defense mechanisms include those involving chlorophyll content and defense enzyme activity [22,23]. That is, virus-infected plants develop strong physiological and biochemical alterations. Photosynthesis is a special, and most basic, life process of green plants providing them with necessary growth and energy [24,25]. Chlorophyll is the photosynthetic pigment of green plants whose content is closely related to photosynthesis, the extent of bacterial infection in plants leading to proliferation and destruction of the plant chloroplasts, and factors retarding the synthesis of chlorophyll causing leaf chlorosis [26,27]. Meanwhile, the increased levels of total chlorophyll were able to improve photosynthesis to enhance plant defense against viral infection [28,29,30]. Therefore, the chlorophyll contents, shown in Figure 4, demonstrate that compound **7t** may increase chlorophyll content to improve photosynthesis, and thereby enhance plant host resistance to viral diseases.

Disease resistance in plants is associated with activation of a wide array of defense responses that slow down or halt infection at certain stages of the host–pathogen interaction. The defense mechanisms include pre-existing physical and chemical barriers that interfere with pathogen establishment. Other methods of protection rely on inducible defense responses in the form of enzymes that are activated upon infection or plant activators [31,32,33,34,35]. The interaction between the pathogen or plant activators and the host plant induces some changes in primary activity of enzymes, particularly SOD, POD, CAT, and PAL, etc. SOD, a key enzyme that resists biological oxidation in the plant, catalyzes the reduction of superoxide anions (O^2−^) to hydrogen peroxide (H_2_O_2_). The diminished capacity for O^2−^ removal ensues decreased ability of progeria cells to minimize oxidative damage and may be a key factor in disease. It plays a critical role in the defense of cells against the toxic effects of oxygen radicals [36]. Antioxidant defense machinery protects plant cells from oxidative damage induced by reactive oxygen species (ROS) to reflect the state of photosynthesis. POD can induce the biosynthetic pathway of salicylic acid (SA), lignin, and phytoalexins, which can activate systemic acquired resistance (SAR) and promote cell-wall reinforcement and pathogen inhibition [37,38]. CAT can catalyze decomposition of hydrogen peroxide to water and oxygen. This process can protect cells against oxidative damage from ROS [39]. PAL is a catalytic enzyme involved in biosynthesis of phenylpropanoids to cinnamic acid, which can produce SA for defense against pathogens [40]. Therefore, we analyzed defensive enzyme activities of **7t**-treated tobacco, the results of defensive enzyme activity assay, shown in Figure 5. demonstrate that compound **7t** may improve disease resistance of tobacco through induction defensive responses in the form of enzymes.

## 4. Materials and Methods

### 4.1. General Methods

The melting points of the products were determined on an XT-4 binocular microscope (Beijing Tech Instrument Co., Beijing, China) and were not corrected. The IR spectra were recorded on a Bruker VECTOR 22 spectrometer (Bruker Optics Inc. Billerica, MA, USA) in KBr disk. ^1^H NMR and ^13^C NMR (solvent DMSO-**d*_6_*) spectral analyses were performed on a JEOL-ECX 500 NMR spectrometer (JEOL Ltd., Tokyo, Japan) at room temperature using tetramethylsilane (TMS) as an internal standard. Elemental analysis was performed on an Elementar Vario-III CHN analyzer (Elementar, Frankfurt, Germany). Mass spectral studies were conducted on an Agilent 5973 organic mass spectrometer (Agilent Technologies, Palo Alto, Canada). Microwave experiments were carried out using a CEM Discover Labmate microwave apparatus (Discover^®^LabMate instrument, CEM Corporate, Matthews, NC, USA). Analytical TLC was performed on silica gel GF254 (200–300 mesh). All solvents were dried by standard methods in advance and distilled before use.

### 4.2. General Procedure for the Preparation of the Key Intermediate **6**

As shown in Scheme 1, 3-(2,6-dichlorophenyl)-5-methylisoxazole-4-carboxylic acid (**1**, 20 mmol) was added to distillated SOCl_2_ (50 mL) and reacted at 80 ^°^C for 3−5 h to obtain intermediate **2**. Then, intermediate **2** (20 mmol) was added dropwise to a stirred solution of substituted *o*-aminobenzoate (**3**, 20 mmol) in anhydrous THF (100 mL) and TEA (1 mL) and the reaction mixture was stirred at room temperature for 1−2 h. The reaction mixture was poured into cold 5.0% dilute HCl solution (200 mL). The solid obtained was filtered, washed with water, and dried under vacuum to give the crude product which was further recrystallized and dried to give intermediate **4**. A mixture of intermediate **4** (20 mmol) and Ac_2_O (200 mmol) was heated under reflux for 3−4 h. The solvent was removed under reduced pressure and then the residue was washed with water, dried under vacuum, and recrystallized from ethanol to give intermediate **5**. Finally, 80% hydrazine hydrate (12 mmol) was added to a solution of intermediate **5** (10 mmol) in THF (50 mL) and reacted at room temperature for 1−2 h. The solvent was removed under reduced pressure, and the residue was washed with water and anhydrous ethanol to give the crude product, then recrystallized from ethanol and dried under vacuum to give the key intermediate **6**.

### 4.3. Preparation of the Target Compounds **7a−7v**

To a 50 mL round-bottom flask with the intermediate **6** (1.0 mmol) and anhydrous ethanol (5 mL), the solutions of different substituted aromatic aldehydes (1.2 mmol) in anhydrous ethanol (5 mL) with a few drops of glacial acetic acid were added dropwise, and then the round-bottom flask was sealed and placed in the synthetic reactor and irradiated in a microwave at 90 °C and 150 W for 1−2 h. Upon completion of the reaction (monitored by TLC), the mixture was concentrated under vacuum, filtered, and recrystallized from a mixture of ethanol and DMF (1:1 in volume) to obtain the target compounds **7a**−**7v**.

### 4.4. In Vivo Antiviral Activity Test

#### 4.4.1. Purification of TMV and CMV

The TMV and CMV viruses, multiplied in *Nicotiana tabacum* (*N. tabacum*) cv. K326, were purified by Gooding’s and Zhou’s methods, respectively [41,42]. The absorbance values were estimated at 260 nm by using an ultraviolet spectrophotometer.

Virus concentration (mg/L) = (A_260_ × dilution ratio)/*E*^0.1%^_1 cm_^260 nm^, where *E* represents the extinction coefficient for TMV, *E*^0.1%^_1 cm_^260 nm^ is 3.1.

#### 4.4.2. Curative Activity of the Target Compounds against TMV In Vivo

Growing *Nicotiana tabacum* L. (*N. tabacum* L.) leaves of the same age were selected and inoculated with TMV (concentration of 6 × 10^−3^ mg/L) by dipping and brushing the whole leaves, which had previously been scattered with silicon carbide. The leaves were washed with water after inoculation for 0.5 h. Then, the solutions of the target compounds were smeared on the left side of the leaves, respectively, instead, the solvent was smeared on the right side as the control. Finally, the number of local lesions was counted and recorded 3–4 d after inoculation. All target compounds were tested with three repetitions to ensure the veracity of the results.

#### 4.4.3. Protection Activity of the Target Compounds against TMV In Vivo

The target compounds solutions were first smeared on the left side of the *N. tabacum* L. leaves, and the solvents were smeared on the right side as the control sample. After 12 h, the crude TMV (concentration of 6 × 10^−3^ mg/L) purified from the leaves of *N. tabacum* cv. K326 was inoculated on whole leaves, which had previously been scattered with silicon carbide. After 0.5 h, the leaves were washed with water and then dried in room temperature. The number of local lesions was recorded 3–4 d after inoculation. All target compounds were tested with three repetitions to ensure the veracity of the results.

#### 4.4.4. Inactivation Activity of the Target Compounds against TMV In Vivo

The virus was inhibited by mixing with the same volume of the compound solution for 0.5 h. The right side of the *N. tabacum* L. leaves was inoculated with the compound solvent, and the virus (concentration of 6 × 10^−3^ mg/L) inoculated on the left side was used as the control. All of the leaves had been previously scattered with silicon carbide. The number of local lesions was recorded 3–4 d after inoculation. All target compounds were tested with three repetitions to ensure the veracity of the results.

#### 4.4.5. Curative Activity of the Target Compounds against CMV In Vivo

The leaves were selected from *Chenopodium amaranticolor* (*C. amaranticolor*) plants of the same age. Crude CMV, at the same concentration (6 × 10^−3^ mg/L), was inoculated with a brush on whole leaves, which had previously been sprinkled with silicon carbide. Subsequently, the leaves were washed with water and dried after inoculation for 0.5 h. The compound solution was then smeared on the right side, and the solvent was smeared on the left side as the control. The number of local lesions was recorded 3–4 d after inoculation. All target compounds were tested with three repetitions to ensure the veracity of the results.

#### 4.4.6. Protection Activity of the Target Compounds against CMV In Vivo

The leaves of *C. amaranticolor* plants of the same age were selected. The compound solution was smeared on the right side of the leaves. Meanwhile, the solvent was smeared on the left side as the control. After 12 h, crude CMV, at the same concentration (6 × 10^−3^ mg/L), was inoculated with a brush on whole leaves, which had previously been sprinkled with silicon carbide. The number of local lesions was then recorded 3–4 d after inoculation. All target compounds were tested with three repetitions to ensure the veracity of the results.

#### 4.4.7. Inactivation Activity of the Target Compounds against CMV In Vivo

The virus was inhibited by mixing with the same volume of the compound solution for 0.5 h. The right side of *C. amaranticolor* leaves was inoculated with the mixture and CMV, diluted to a suitable concentration (6 × 10^−3^ mg/L), inoculated on the left side as the control. All leaves had previously been sprinkled with silicon carbide. The number of local lesions was then recorded 3–4 d after inoculation. All target compounds were tested with three repetitions to ensure the veracity of the results.

The inhibition rates of the target compounds were calculated according to the following formula (“av.” means average):

Inhibition rate (%) = [(av. local lesion number of control (not treated with compound) – av. local lesion number smeared with compound)/av. local lesion number of control (not treated with compound)] × 100%

On the basis of the preliminary biological activity, the EC_50_ values were also determined and calculated via the software SPSS 17.0 (SPSS, Chicago, IL). Meanwhile, statistical analysis was performed by Student’s *t*-test via the software SPSS 17.0 (SPSS, Chicago, IL), “*” and “**” indicate the values of antiviral activity of the target compounds with significant difference compared with those of NNM at *p* < 0.05 and *p* < 0.01, respectively.

### 4.5. Physiological and Biochemical Analysis

#### 4.5.1. Plant Growth and Sample Collection

Similarly grown *N. tabacum L.* K326 was selected at the 7-leaf stage, and compound **7t** solution, at a concentration of 500 mg/L, was smeared on whole leaves. Meanwhile, solvent and NNM were used as negative control (CK) and positive control, respectively. After smearing for 12 h, the leaves were inoculated with the TMV virus and then cultivated in a greenhouse. Four treatments were adopted: CK, CK + TMV, NNM + TMV, and **7t** + TMV. Tissue samples were collected at 1, 3, 5, and 7 d after inoculation treatment for assays on chlorophyll content and defensive enzyme activities assay. Measurements were performed in triplicate.

#### 4.5.2. Chlorophyll Content Test

In this study, chlorophyll contents of samples were measured using a modified reported method [43]. Test samples in triplicate of each treatment were sliced into small uniform pieces by a hole puncher while avoiding the midrib. Samples (50 mg) were placed in 5 mL of a cold solution of a 1:1 mixture of 85% acetone and 85% ethanol (*v*/*v*). Samples were homogenized, incubated for 0.5 h at 35 °C, and centrifuged for 15 min at 6500 rpm. Absorbance spectra were recorded at 663 and 645 nm for C_a_ and C_b_, respectively, against a solution as reference. C_a_, C_b_, and C_t_ were calculated by the following equations:

C_a_ (mg/L) = 0.0127OD_663_ – 0.00269OD_645_ (1)

C_b_ (mg/L) = 0.0229OD_645_ – 0.00468OD_663_ (2)

C_t_ (mg/L) = C_a_ + C_b_ (3)

#### 4.5.3. Determination of Defensive Enzyme Activity

Activities of SOD, POD, CAT, and PAL were measured and calculated with enzyme assay reagent kits in accordance with the manufacturer’s instructions (Suzhou Comin Bioengineering Institute, Suzhou, China).

## 5. Conclusions

In this study, a total of 22 novel isoxazole-amide derivatives containing an acylhydrazone moiety were designed and synthesized. Antiviral activity results showed that compound **7t** exhibited the best curative, protection, and inactivation activities against TMV and CMV and which were even better than those of NNM. Meanwhile, we also found that compound **7t** could enhance the defense-related enzyme activity and increase the chlorophyll content in tobacco leaves to induce resistance and enhance plant tolerance to TMV infection. Furthermore, according to the requirements of pesticide registration in China, further field studies on the biological efficacies, crop safety, and toxicities of compound **7t** as an antiviral candidate will be performed in our next work.

## Supplementary Materials

The following are available online, Table S1. The preliminary in vivo antiviral activities against TMV and CMV of the target compounds **7a–7v** at 500 mg/L. Table S2. The EC_50_ values of the target compounds **7a–7v** against TMV in vivo. Table S3. The EC_50_ values of the target compounds **7a–7v** against CMV in vivo. Figure S1. The antiviral activity of compound **7t** against TMV at 500 mg/L. Left: smeared with solvent; Right: smeared with compound **7t** or NNM. Figure S2. The antiviral activity of compound **7t** against CMV at 500 mg/L. Left: smeared with solvent; Right: smeared with compound **7t** or NNM.
