# Supplementary material for: Design, Synthesis, and Bioactivity Evaluation of Novel Isoxazole-Amide Derivatives Containing an Acylhydrazone Moiety as New Active Antiviral Agents
Source: Molecules. 2019 Oct 19;24(20):3766. doi: 10.3390/molecules24203766 (PMC6832660; doi:10.3390/molecules24203766)
Supplement: Supplementary file 1 [file molecules-24-03766-s001.pdf]

Design, Synthesis and Bioactivity Evaluation of Novel Isoxazole-Amide Derivatives Containing an Acylhydrazone Moiety as New Active Antiviral Agents

Zai-Bo Yang [1,*,†], Pei Li [1,2,*,†] and Yin-Ju He [1]

[1] School of Chemistry and Chemical Engineering, Qiannan Normal University for Nationalities, Duyun 558000, China; heyinju2007@163.com

[2] Qiandongnan Engineering and Technology Research Center for Comprehensive Utilization of National Medicine, Kaili University, Kaili 556011, China

[*] Correspondence: yzb1976110@sohu.com (Z-B.Y.); pl19890627@126.com (P.L.); Tel.: +86(854)873-7046

[†] These authors contributed equally to this work.

Table S1. The preliminary in vivo antiviral activities against TMV and CMV of the target compounds **7a–7v** at 500 mg/L.

Compounds	TMV			CMV		
	Curative activity (%)	Protection activity (%)	Inactivation activity (%)	Curative activity (%)	Protection activity (%)	Inactivation activity (%)
**7a**	48.5 ± 3.0	54.2 ± 3.1	85.6 ± 1.7	38.4 ± 1.2	47.6 ± 1.3	69.9 ± 1.5
**7b**	40.8 ± 2.1	46.5 ± 5.0	83.4 ± 2.1	33.6 ± 1.4	44.7 ± 1.8	65.8 ± 1.7
**7c**	39.4 ± 1.3	59.4 ± 2.2	87.7 ± 2.3	37.9 ± 1.9	54.6 ± 2.1	72.6 ± 2.4
**7d**	62.9 ± 1.2	67.2 ± 3.0	93.6 ± 3.3	56.7 ± 2.1	63.8 ± 2.5	91.4 ± 2.6
**7e**	28.2 ± 3.1	39.8 ± 2.1	55.1 ± 2.9	14.4 ± 1.3	24.6 ± 1.5	42.3 ± 1.2
**7f**	36.7 ± 1.4	50.2 ± 4.2	78.8 ± 1.6	33.5 ± 1.6	43.6 ± 2.3	76.9 ± 2.1
**7g**	35.6 ± 2.2	42.4 ± 1.7	74.7 ± 1.8	30.7 ± 1.8	40.5 ± 1.4	70.8 ± 1.9
**7h**	64.5 ± 1.2	69.6 ± 2.6	94.1 ± 1.2	58.8 ± 2.2	65.4 ± 2.5	92.0 ± 2.6
**7i**	50.4 ± 1.2	58.2 ± 2.3	86.4 ± 1.9	47.3 ± 1.6	54.7 ± 2.1	80.7 ± 1.8
**7j**	55.4 ± 1.3	65.3 ± 1.1	92.6 ± 3.2	51.6 ± 1.7	57.5 ± 1.6	81.8 ± 2.7
**7k**	47.5 ± 1.1	56.4 ± 2.3	85.5 ± 3.7	48.3 ± 1.5	54.8 ± 2.2	78.4 ± 2.6
**7l**	43.9 ± 4.2	60.4 ± 4.1	89.4 ± 3.3	41.6 ± 1.8	56.2 ± 2.4	75.8 ± 2.3
**7m**	65.1 ± 2.2	69.7 ± 1.0	95.3 ± 2.6	58.6 ± 2.5	64.4 ± 1.7	92.8 ± 2.8
**7n**	36.5 ± 1.0	43.6 ± 1.2	67.5 ± 1.8	26.5 ± 1.1	34.8 ± 1.4	52.9 ± 1.3
**7o**	42.2 ± 4.5	56.3 ± 1.5	84.2 ± 2.1	39.6 ± 2.2	49.7 ± 1.6	75.8 ± 2.5
**7p**	40.9 ± 4.1	49.4 ± 1.0	78.2 ± 1.4	36.2 ± 2.0	44.8 ± 1.4	69.9 ± 1.8
**7q**	67.0 ± 2.3	72.7 ± 2.2	96.3 ± 1.5	60.8 ± 2.7	67.8 ± 2.9	93.8 ± 1.9
**7r**	56.7 ± 2.1	64.3 ± 2.3	93.3 ± 3.1	57.9 ± 2.5	63.6 ± 2.4	91.9 ± 2.8
**7s**	52.3 ± 1.6	60.3 ± 4.1	89.1 ± 1.3	47.7 ± 1.8	52.2 ± 2.3	80.6 ± 1.5
**7t**	68.8 ± 2.5	74.8 ± 2.6	96.9 ± 2.9	65.9 ± 2.8	70.2 ± 2.5	94.6 ± 2.4
**7u**	40.5 ± 2.2	57.4 ± 2.0	76.9 ± 1.2	36.2 ± 2.1	48.9 ± 2.2	68.7 ± 2.3
**7v**	37.2 ± 3.1	42.5 ± 3.0	67.7 ± 2.8	31.4 ± 1.2	38.6 ± 1.4	62.0 ± 2.5
**NNM**	55.6 ± 1.6	63.8 ± 2.9	92.5 ± 1.3	54.7 ± 3.4	62.6 ± 2.5	91.0 ± 3.0

Table S2. The EC_{50} values of the target compounds **7a–7v** against TMV in vivo.

Compounds	EC_{50} (mg/L)		
	Curative activity	Protection activity	Inactivation activity
**7a**	358.5 ± 3.4	256.5 ± 3.4	97.8 ± 1.6
**7b**	395.8 ± 3.1	324.7 ± 3.2	132.8 ± 1.9
**7c**	356.8 ± 3.2	288.6 ± 2.8	105.6 ± 1.8
**7d**	238.9 ± 1.9	187.2 ± 2.4	46.7 ± 1.2
**7e**	536.3 ± 3.7	429.8 ± 2.9	245.1 ± 2.3
**7f**	370.7 ± 3.5	290.7 ± 3.8	148.9 ± 2.6
**7g**	395.6 ± 3.2	322.6 ± 2.2	171.5 ± 2.1
**7h**	195.5 ± 2.3	172.6 ± 2.8	43.1 ± 1.5
**7i**	516.7 ± 3.2	345.2 ± 2.6	86.4 ± 1.2
**7j**	291.4 ± 3.1	232.3 ± 2.6	52.9 ± 1.5
**7k**	364.5 ± 3.5	276.8 ± 2.9	118.7 ± 1.7
**7l**	336.8 ± 4.7	249.4 ± 4.5	95.8 ± 1.6
**7m**	189.9 ± 2.5	176.5 ± 2.5	44.5 ± 1.5
**7n**	487.6 ± 4.8	361.7 ± 2.7	228.5 ± 2.3
**7o**	368.6 ± 4.3	280.3 ± 3.3	123.7 ± 2.6
**7p**	385.6 ± 4.1	299.8 ± 3.2	146.8 ± 2.5
**7q**	179.6 ± 3.5	169.2 ± 2.9	36.9 ± 1.8
**7r**	266.8 ± 2.9	199.4 ± 3.5	42.7 ± 1.6
**7s**	332.3 ± 3.2	296.5 ± 3.5	80.6 ± 1.4
**7t**	168.5 ± 1.9	157.6 ± 2.1	33.7 ± 1.7

**7u**	417.8 ± 2.8	377.1 ± 2.3	172.4 ± 3.6
**7v**	448.9 ± 3.7	421.6 ± 3.5	197.7 ± 3.8
**NNM**	286.4 ± 2.2	198.2 ± 2.1	46.3 ± 1.9

Table S3. The EC_{50} values of the target compounds **7a–7v** against CMV in vivo.

**Compounds**	EC_{50} (mg/L)		
	Curative activity	Protection activity	Inactivation activity
**7a**	365.2 ± 3.6	338.6 ± 3.2	114.5 ± 1.9
**7b**	378.2 ± 3.8	357.7 ± 3.5	138.2 ± 2.3
**7c**	387.6 ± 3.6	347.4 ± 2.8	147.8 ± 2.3
**7d**	276.5 ± 2.4	247.8 ± 2.9	57.3 ± 1.9
**7e**	547.6 ± 3.5	528.2 ± 2.6	318.6 ± 2.9
**7f**	412.6 ± 3.8	389.3 ± 3.6	193.6 ± 2.9
**7g**	428.9 ± 3.8	406.4 ± 2.9	223.5 ± 3.2
**7h**	226.4 ± 3.5	215.8 ± 2.5	53.7 ± 1.8
**7i**	361.2 ± 3.5	328.7 ± 2.9	108.7 ± 1.3
**7j**	338.7 ± 3.4	288.9 ± 3.5	93.8 ± 1.8
**7k**	355.5 ± 3.9	319.6 ± 2.5	105.4 ± 1.9
**7l**	358.8 ± 4.2	331.5 ± 3.6	129.6 ± 1.8
**7m**	248.5 ± 3.6	198.3 ± 3.2	51.8 ± 1.4
**7n**	519.9 ± 4.7	488.3 ± 4.5	287.8 ± 2.9
**7o**	395.4 ± 3.7	379.8 ± 3.4	169.2 ± 2.9
**7p**	407.4 ± 4.2	389.4 ± 3.6	192.3 ± 2.8
**7q**	227.3 ± 3.2	179.7 ± 2.5	47.6 ± 1.9
**7r**	268.5 ± 2.6	236.9 ± 3.2	56.4 ± 1.4
**7s**	353.7 ± 3.5	342.7 ± 3.6	89.4 ± 1.3
**7t**	197.9 ± 2.8	168.4 ± 2.8	45.8 ± 1.2
**7u**	438.9 ± 3.6	422.8 ± 2.9	226.8 ± 3.9
**7v**	456.7 ± 3.9	438.9 ± 3.9	234.6 ± 3.6
**NNM**	297.3 ± 1.4	263.4 ± 2.5	62.8 ± 1.8

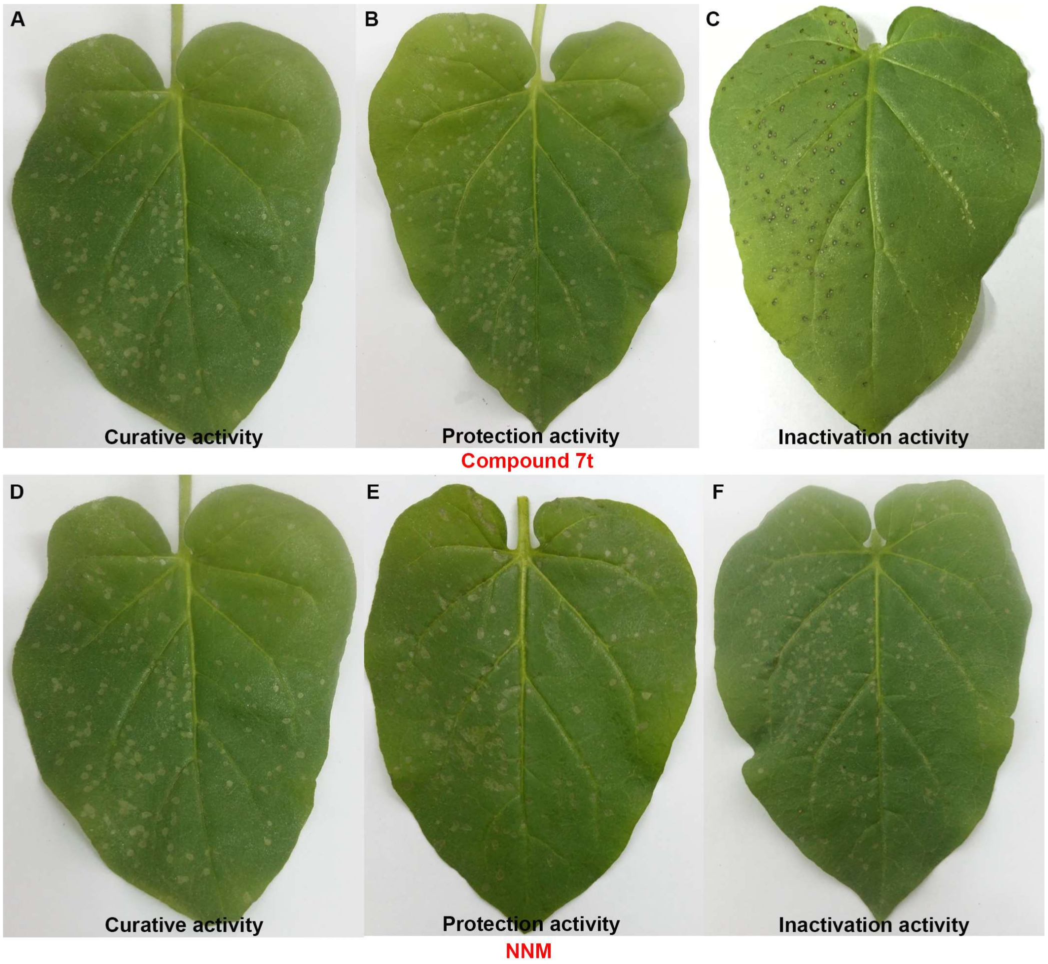

Figure S1. The antiviral activity of compound **7t** against TMV at 500 mg/L. Left: smeared with solvent; Right: smeared with compound **7t** or NNM.

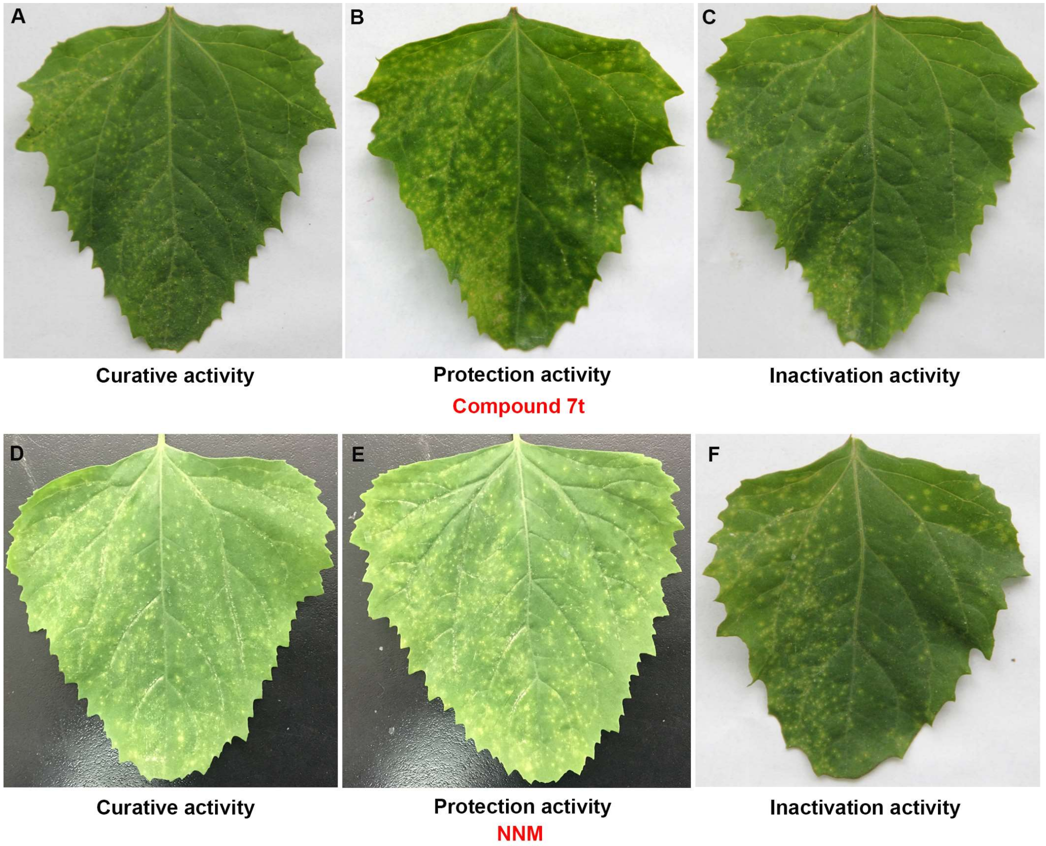

Figure S2. The antiviral activity of compound **7t** against CMV at 500 mg/L. Left: smeared with solvent; Right: smeared with compound **7t** or NNM.

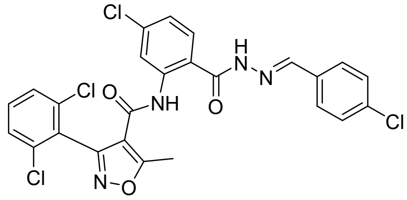

7a

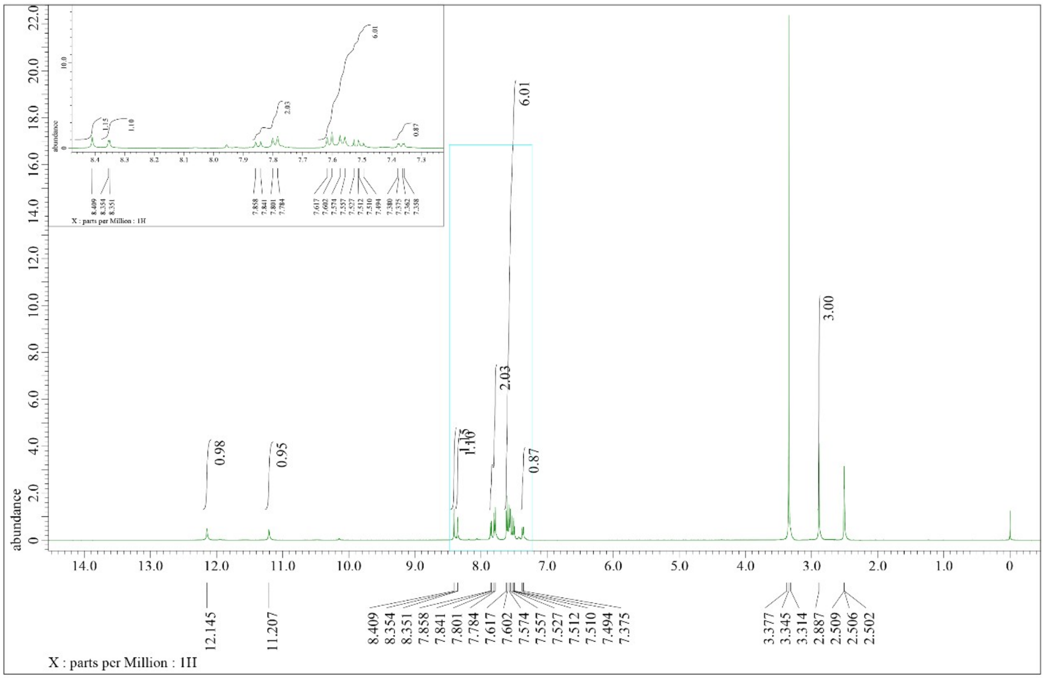

^{1}H NMR of Compound 7a

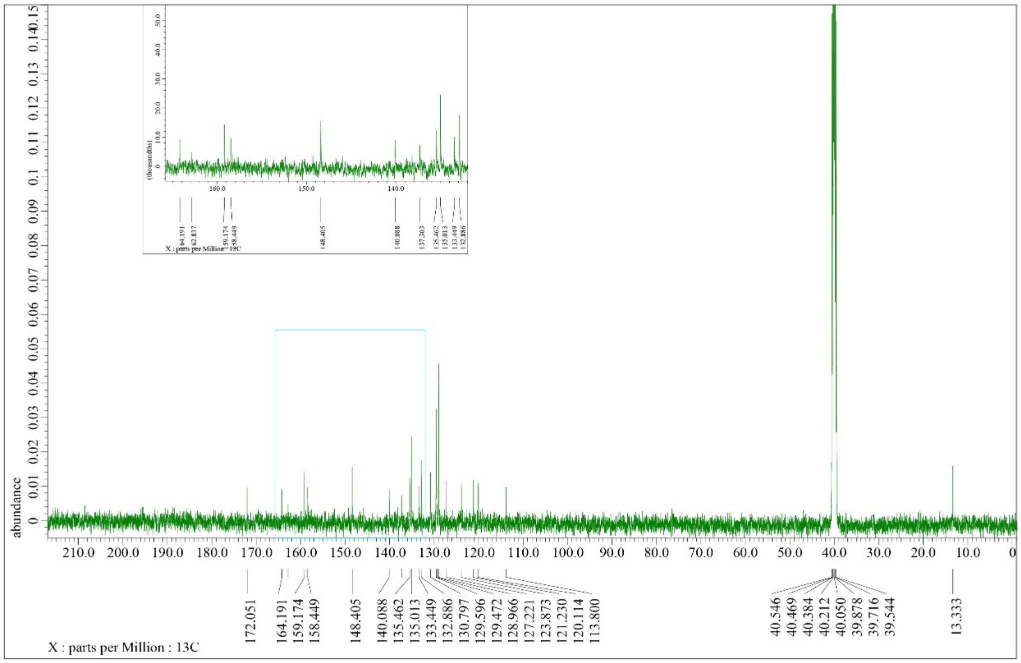

^{13}C NMR of Compound 7a

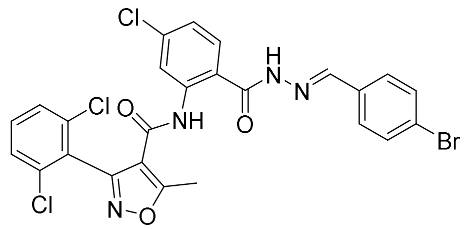

4b

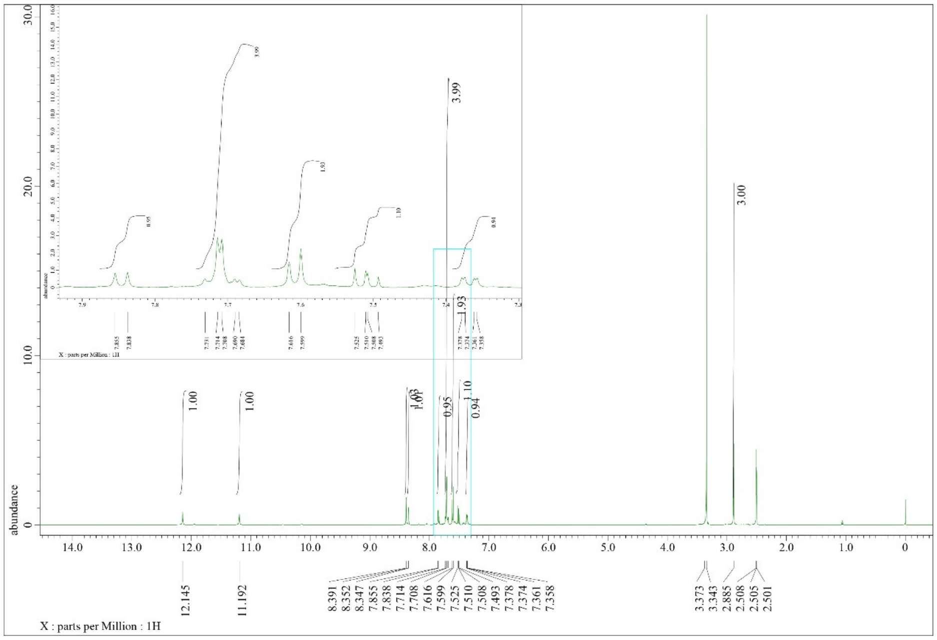

^{1}H NMR of Compound 7b

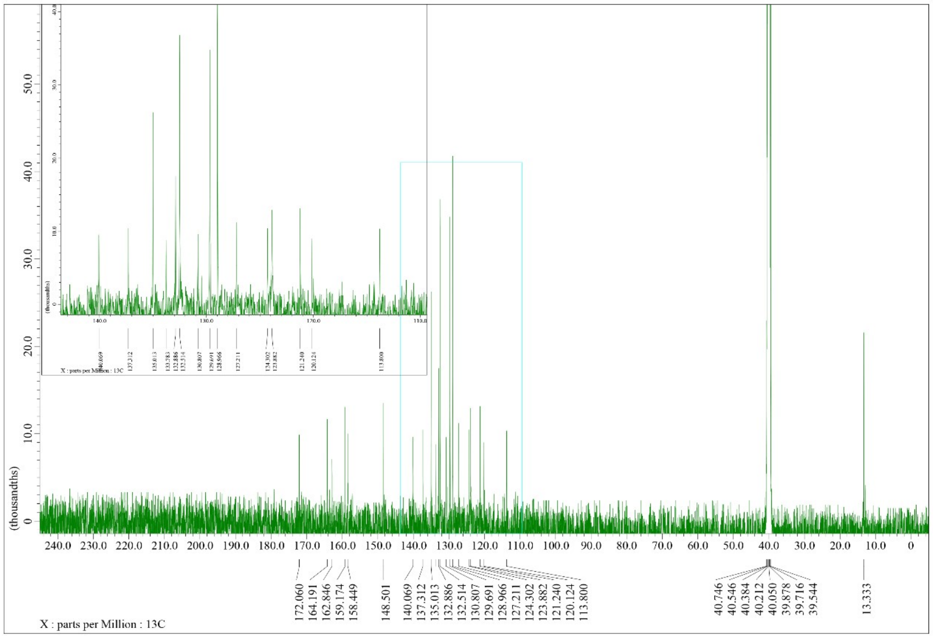

^{13}C NMR of Compound 7b

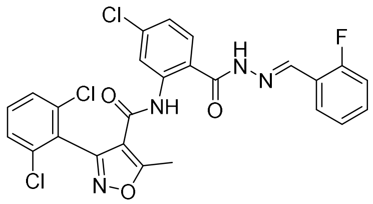

7c

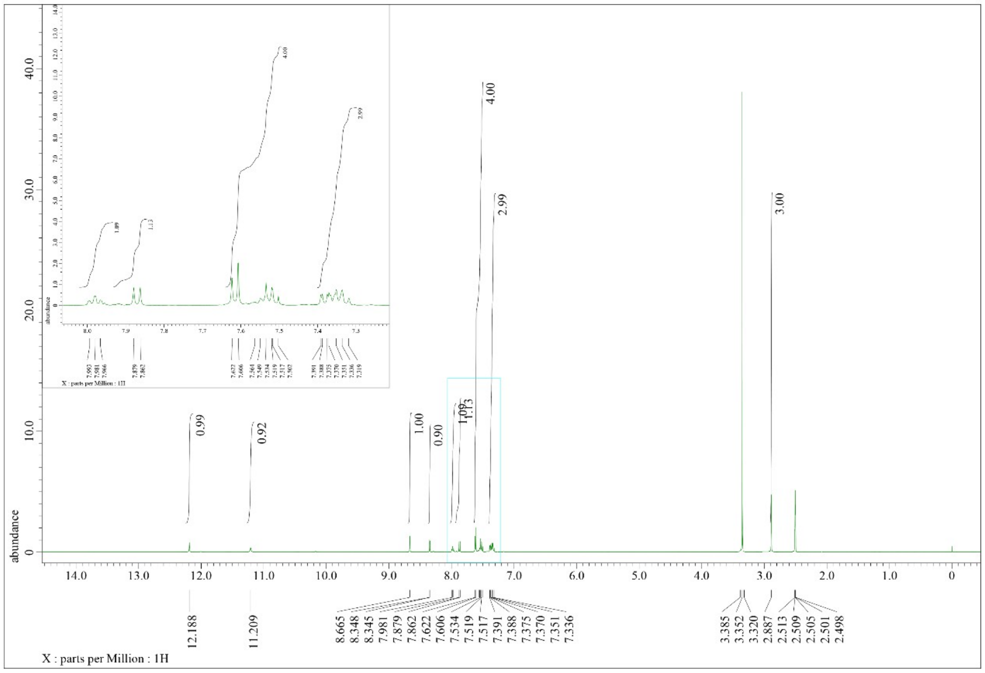

^{1}H NMR of Compound 7c

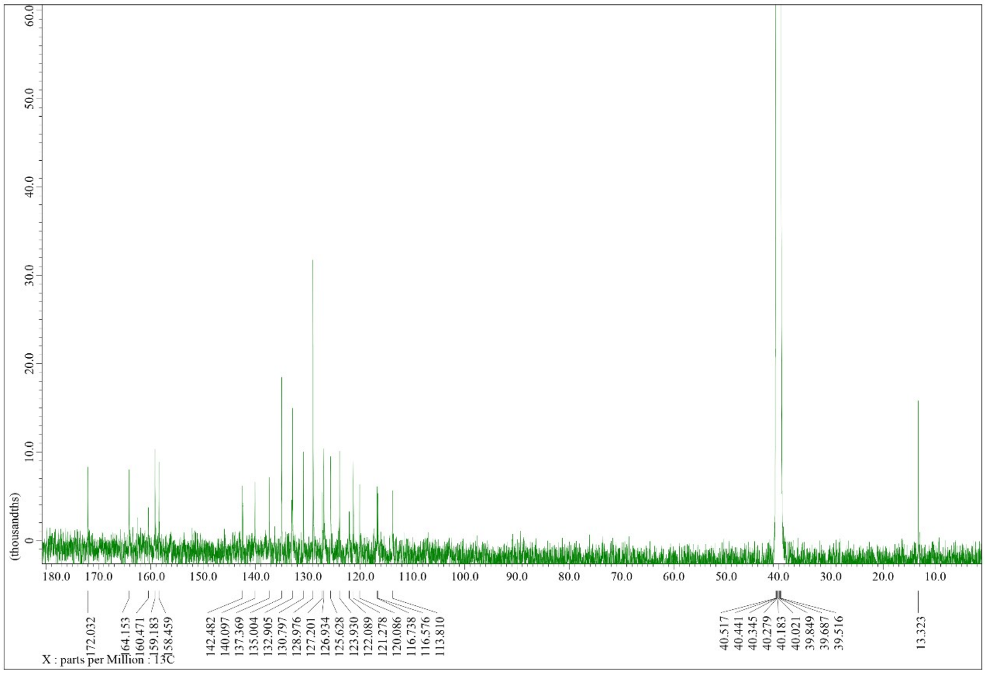

^{13}C NMR of Compound 7c

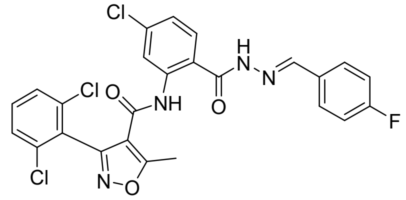

7d

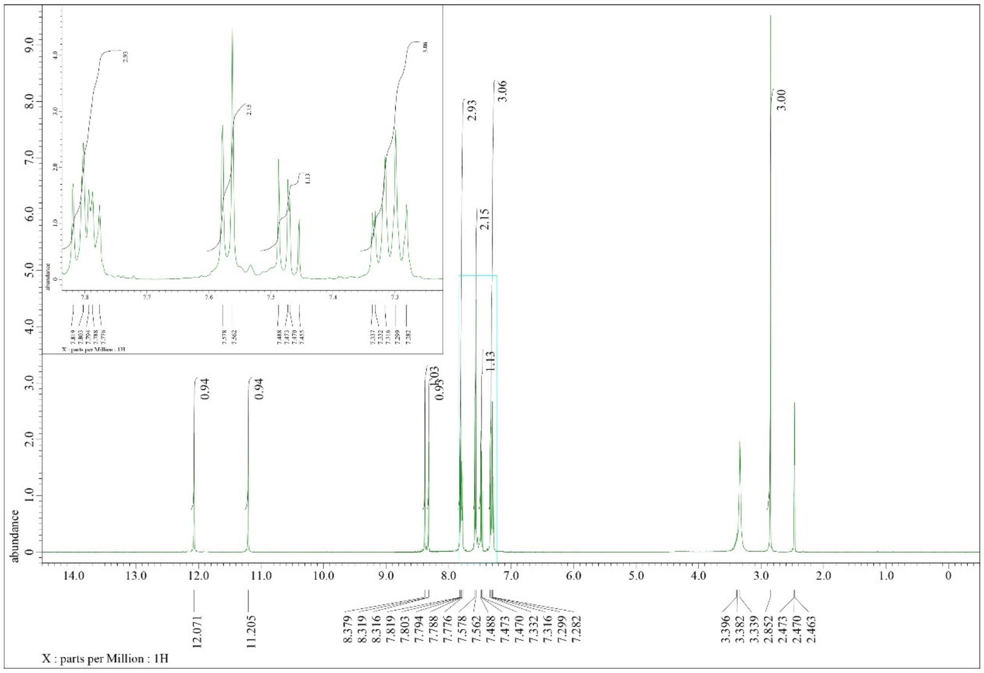

^{1}H NMR of Compound 7d

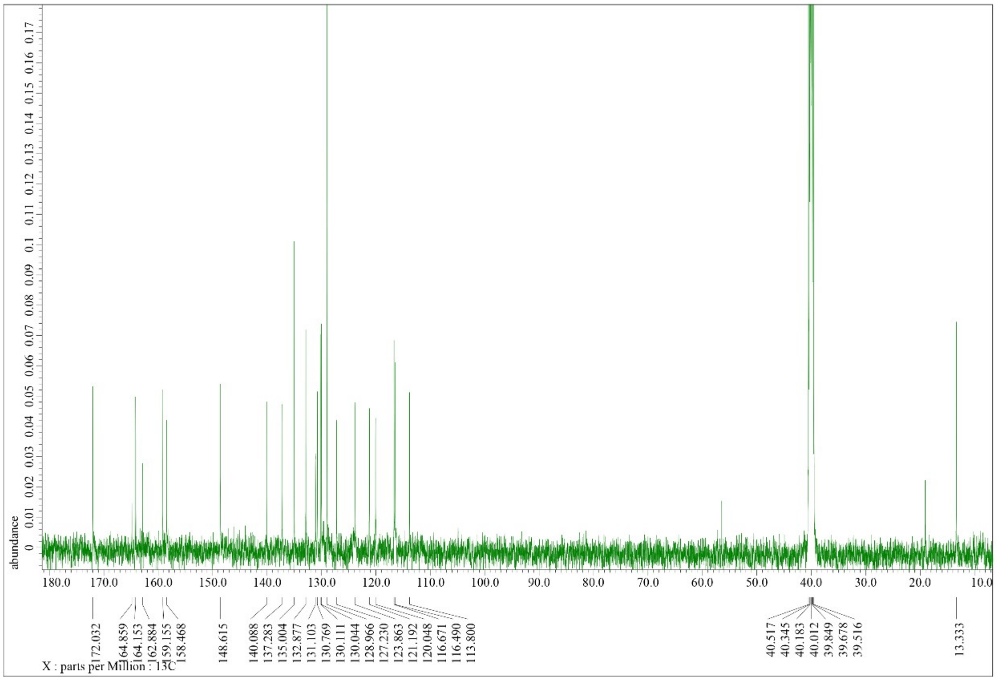

^{13}C NMR of Compound 7d

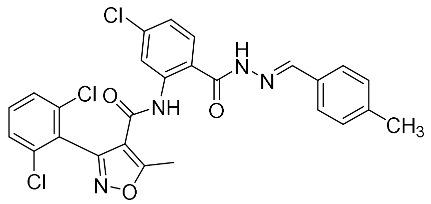

7e

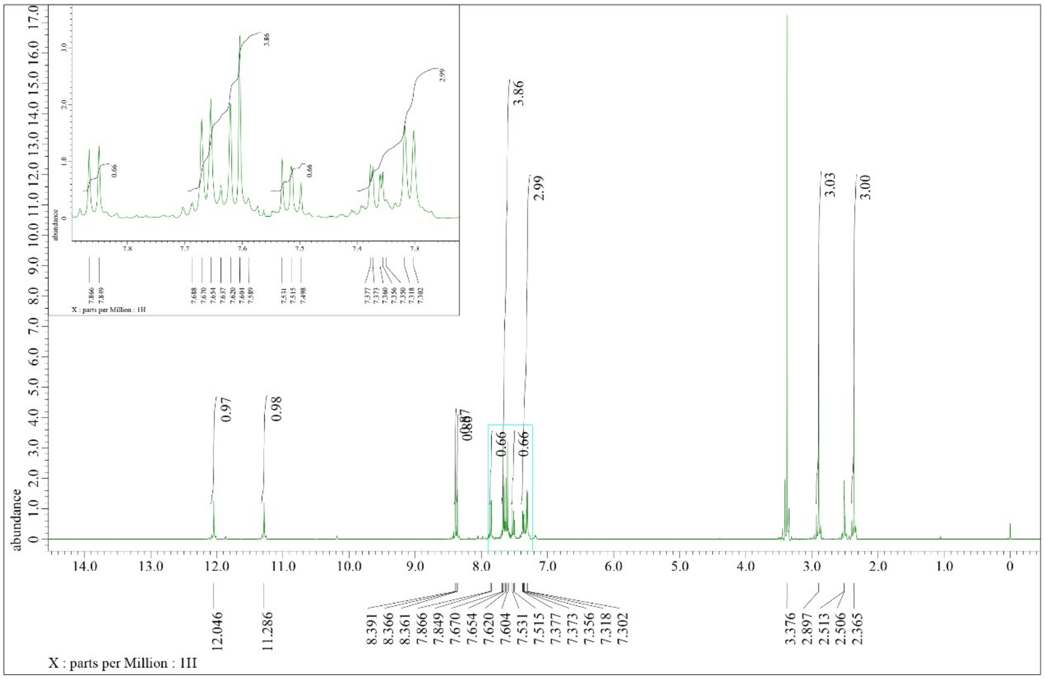

^{1}H NMR of Compound 7e

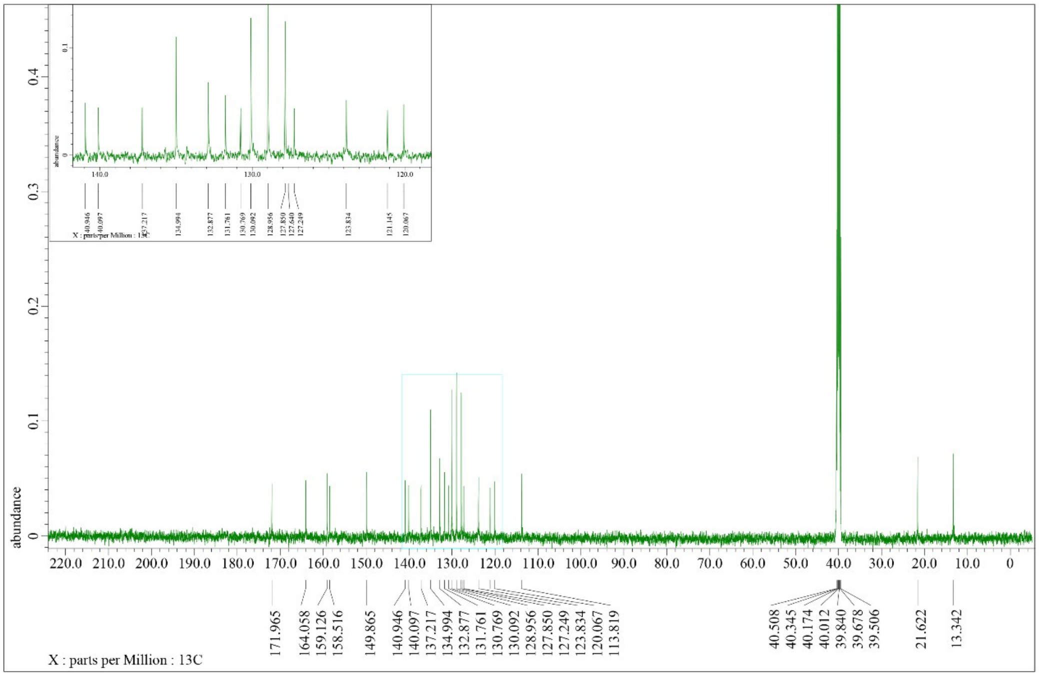

^{13}C NMR of Compound 7e

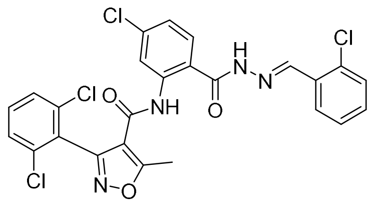

7f

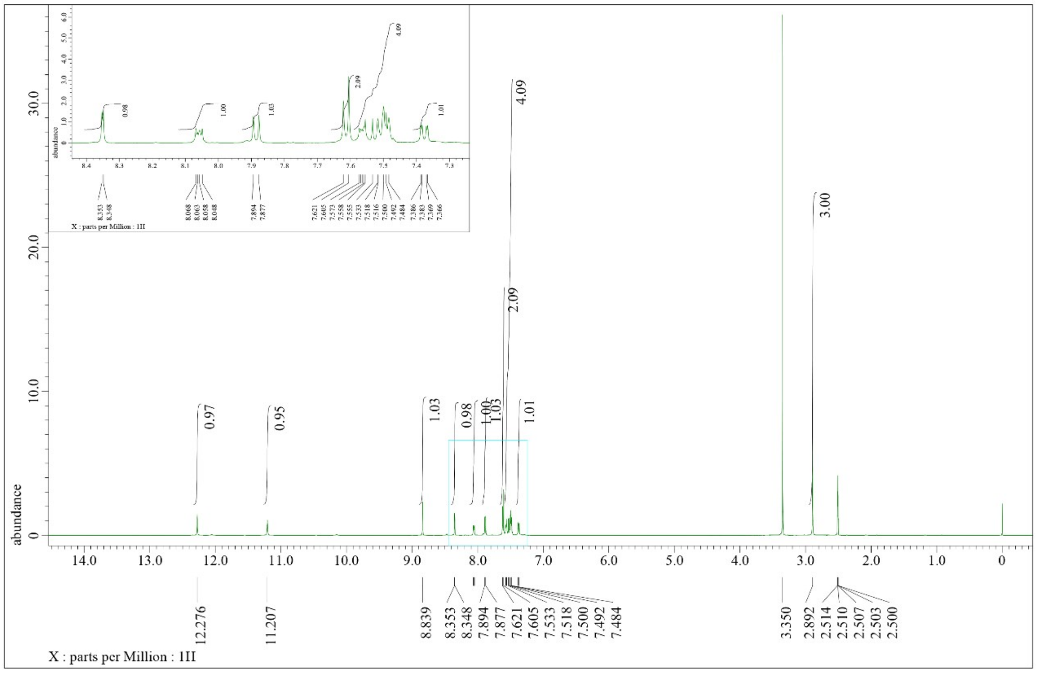

^{1}H NMR of Compound 7f

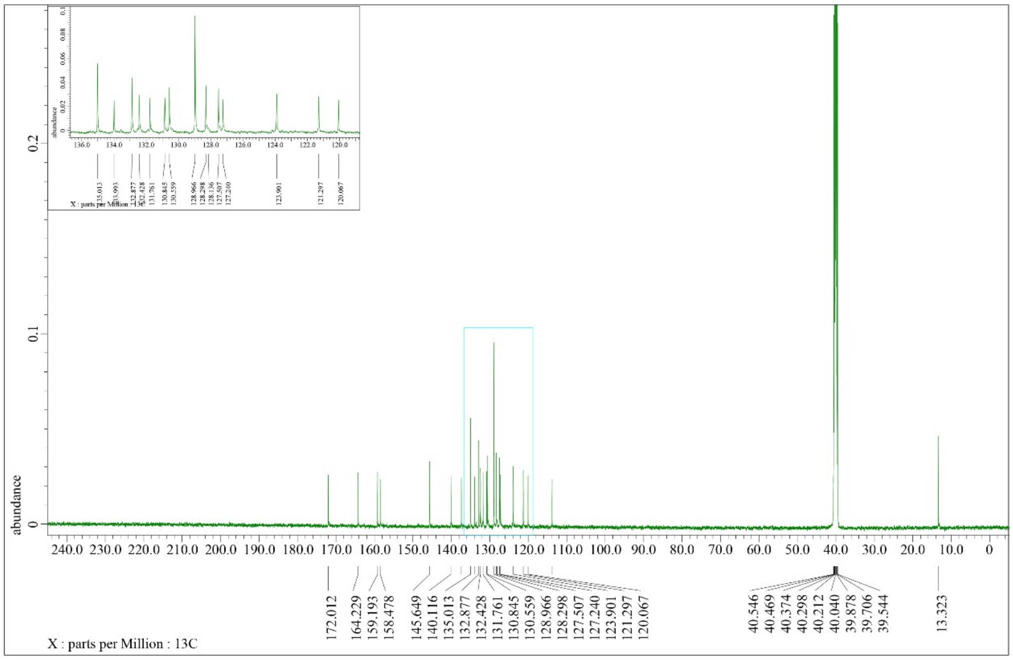

^{13}C NMR of Compound 7f

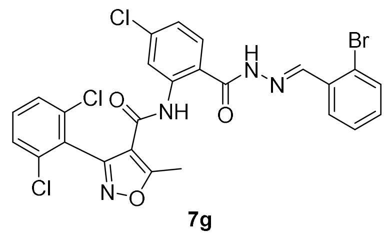

7g

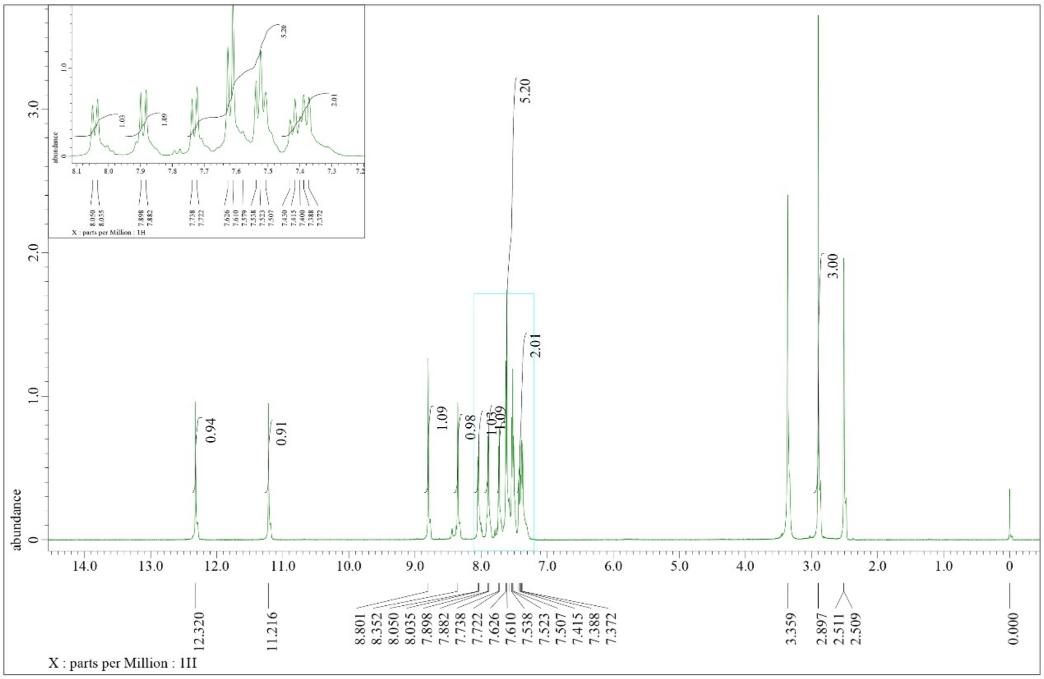

^{1}H NMR of Compound 7g

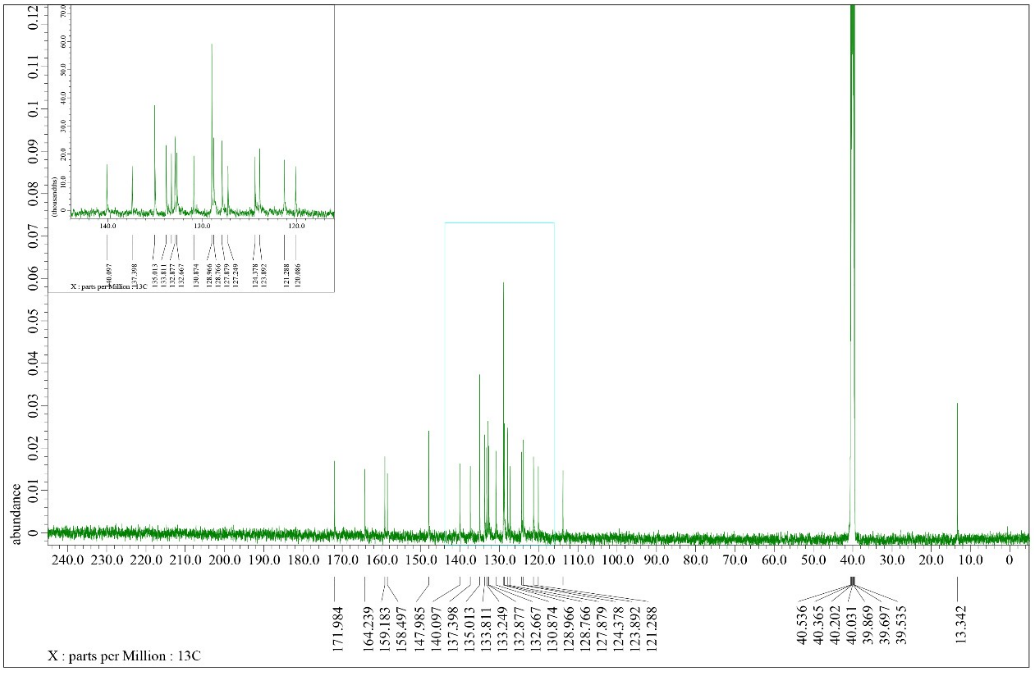

^{13}C NMR of Compound 7g

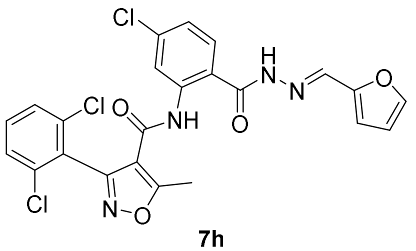

7h

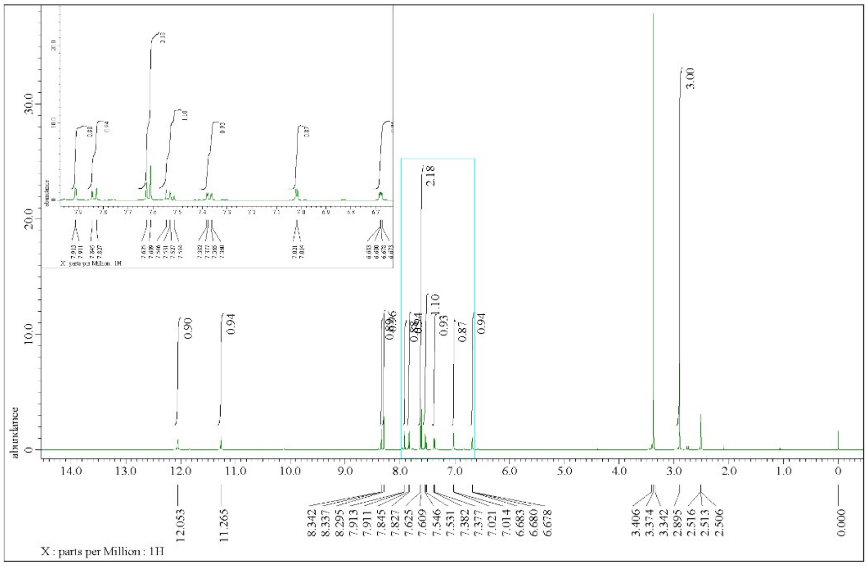

^{1}H NMR of Compound 7h

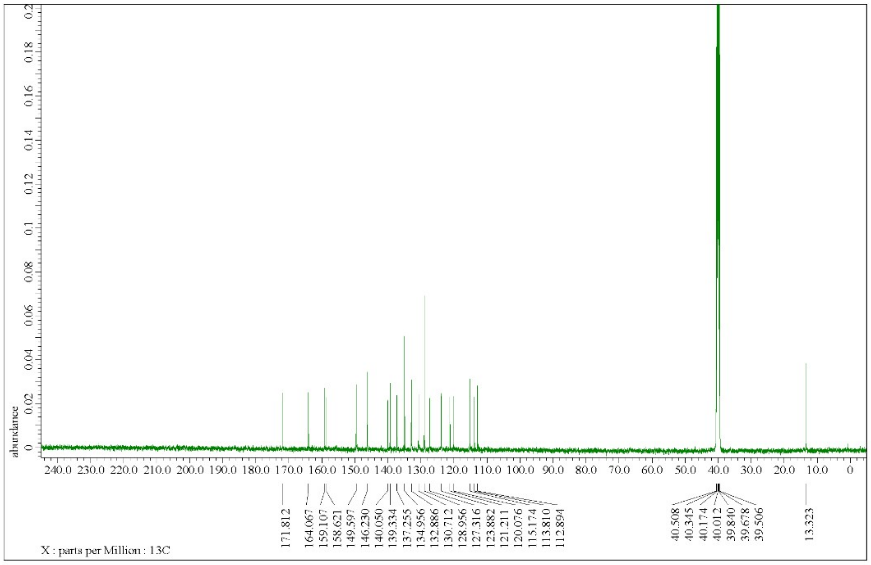

^{13}C NMR of Compound 7h

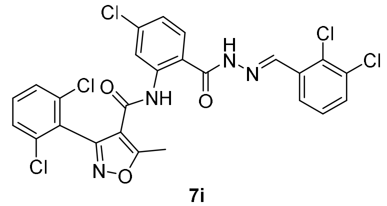

7i

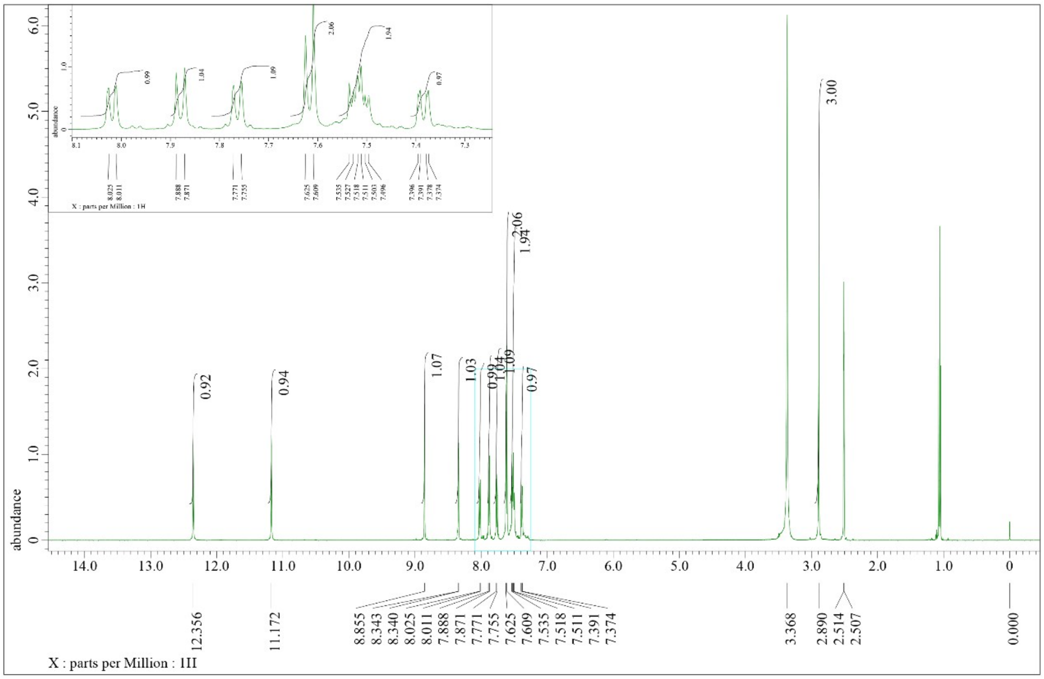

^{1}H NMR of Compound 7i

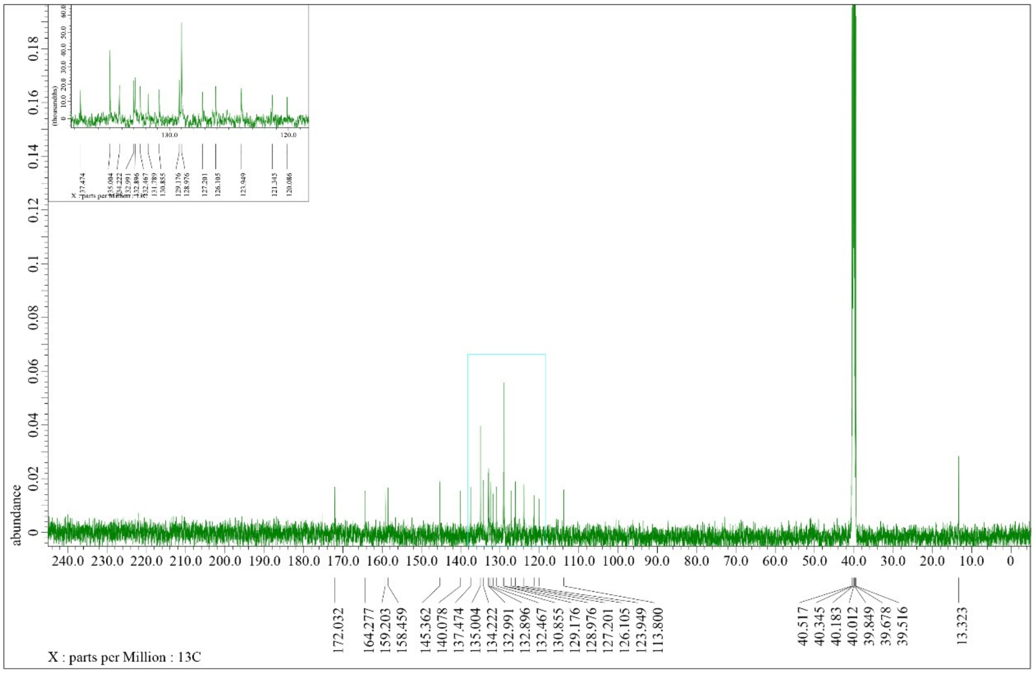

^{13}C NMR of Compound 7i

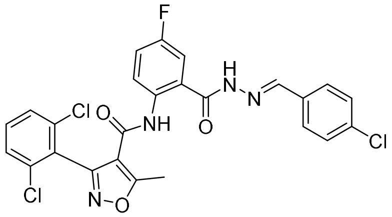

7j

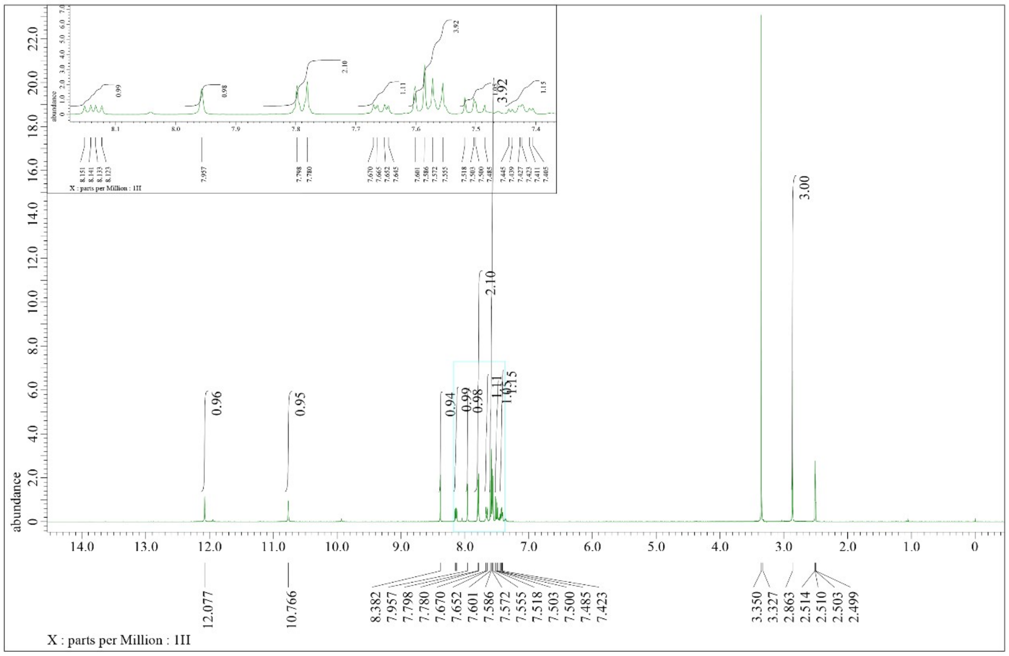

^{1}H NMR of Compound 7j

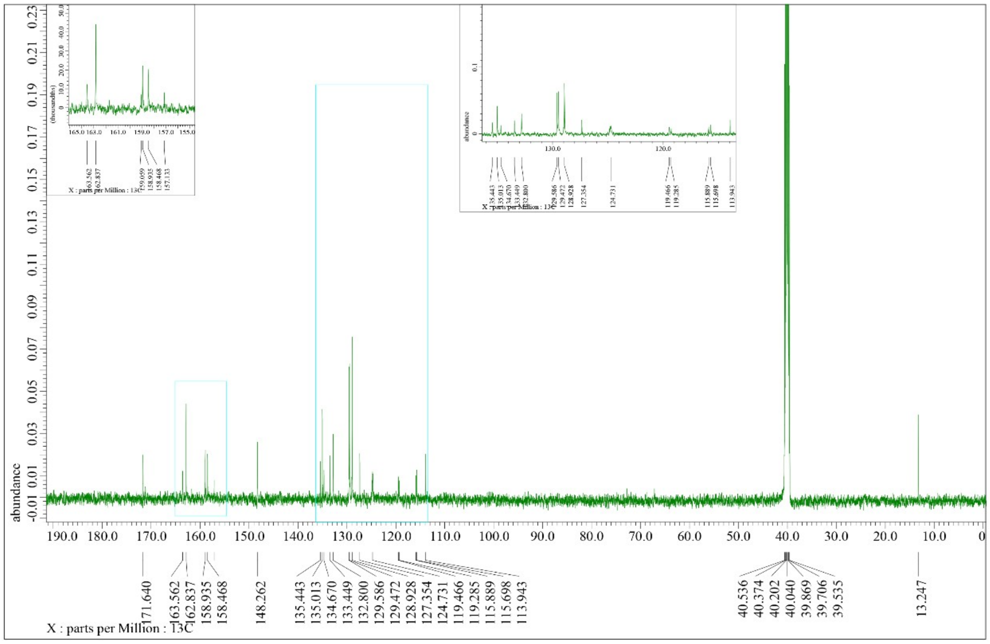

^{13}C NMR of Compound 7j

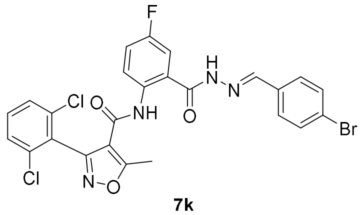

7k

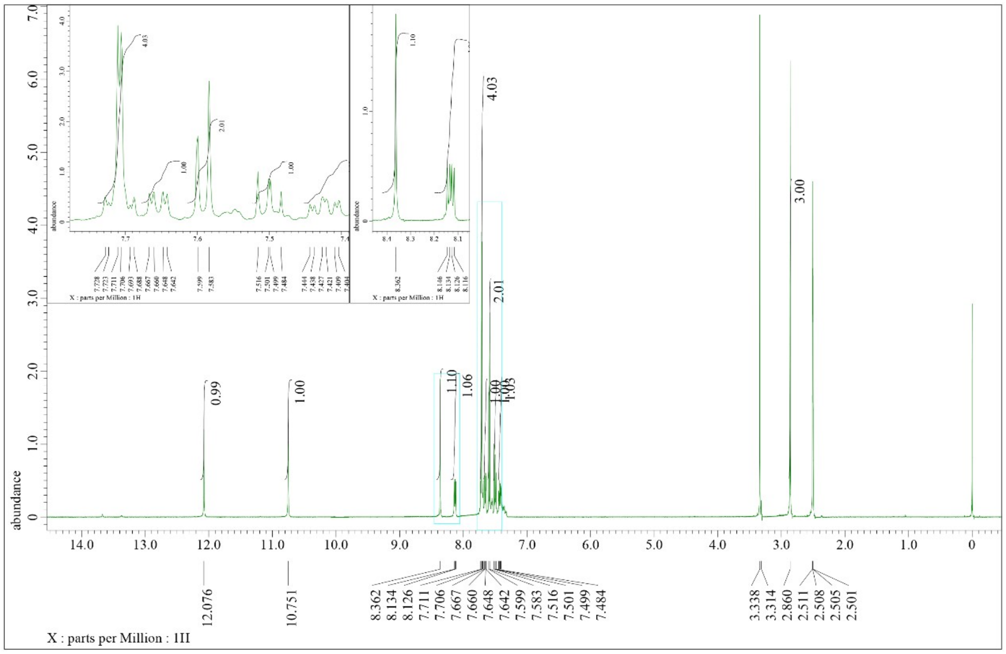

^{1}H NMR of Compound 7k

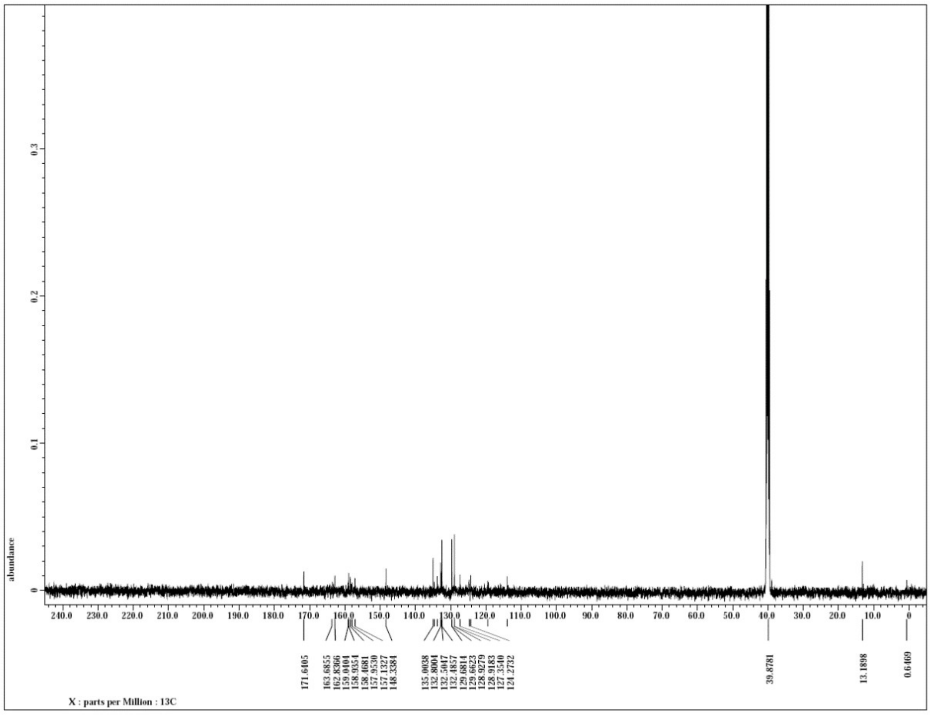

^{13}C NMR of Compound 7k

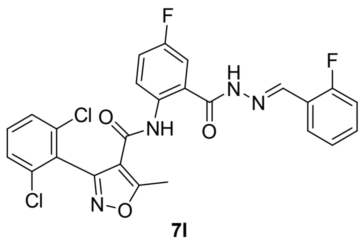

7l

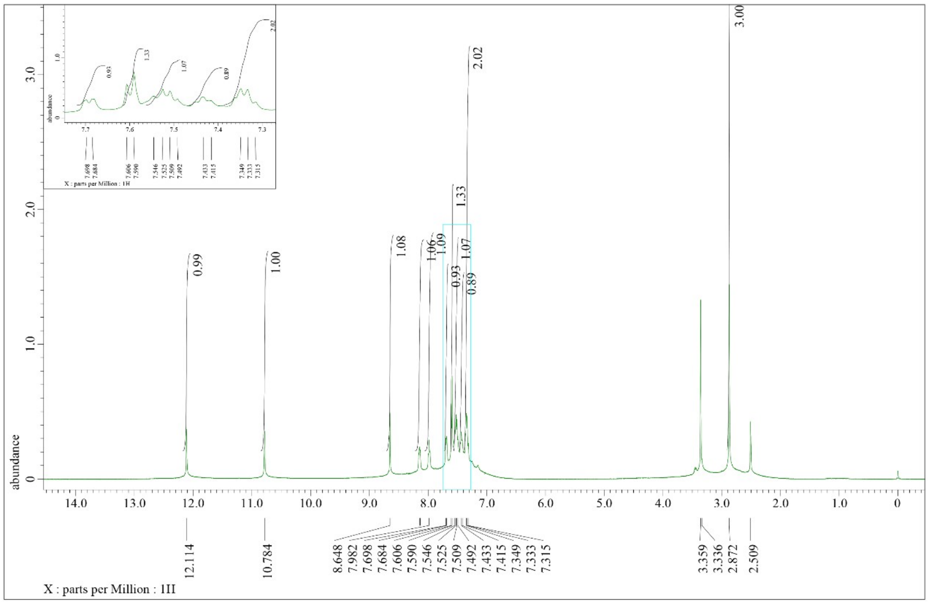

^{1}H NMR of Compound 7l

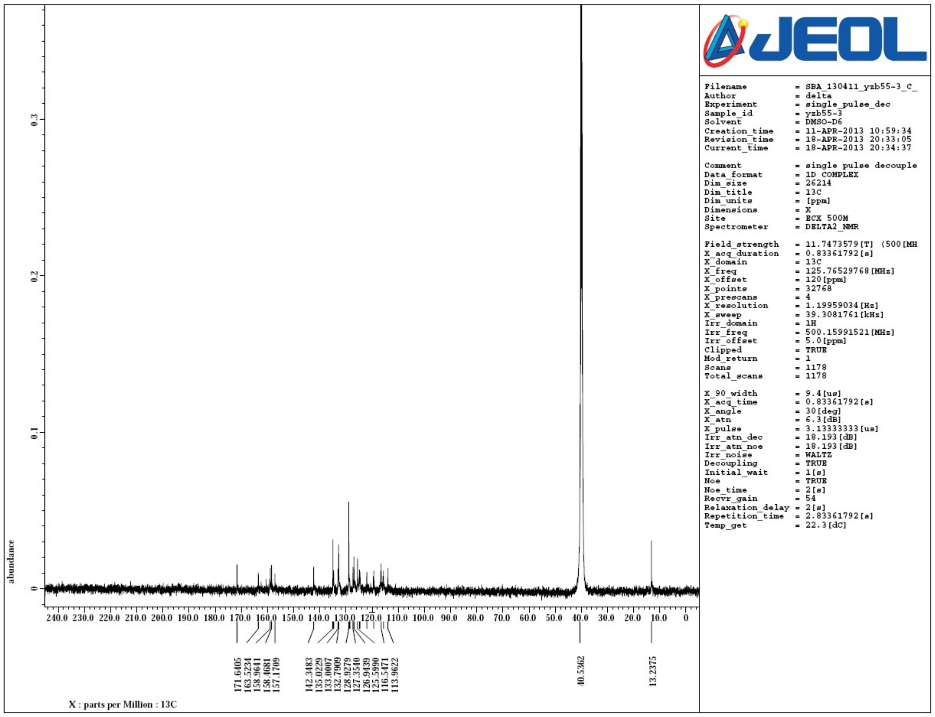

^{13}C NMR of Compound 7l

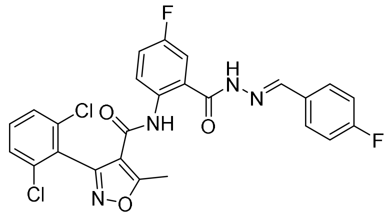

7m

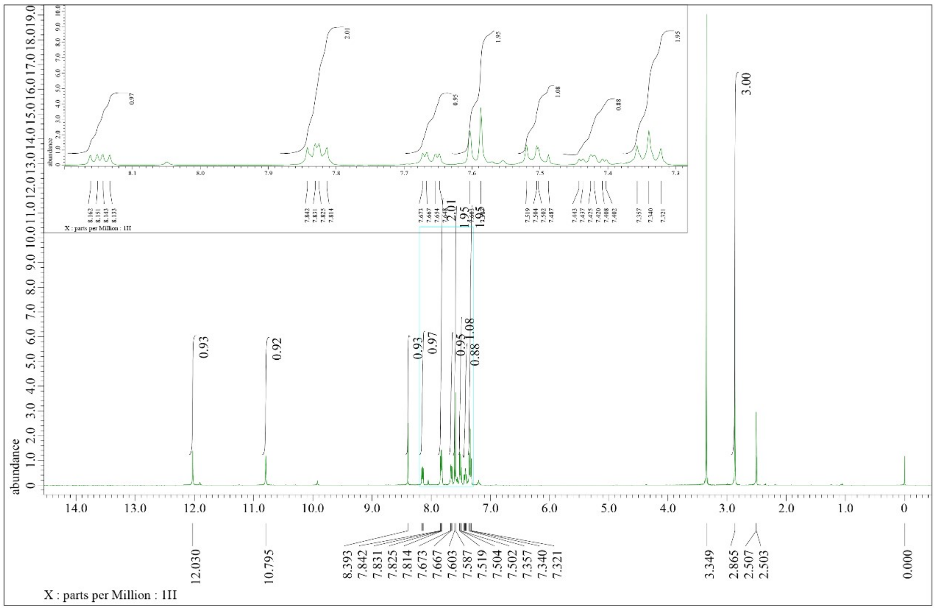

^{1}H NMR of Compound 7m

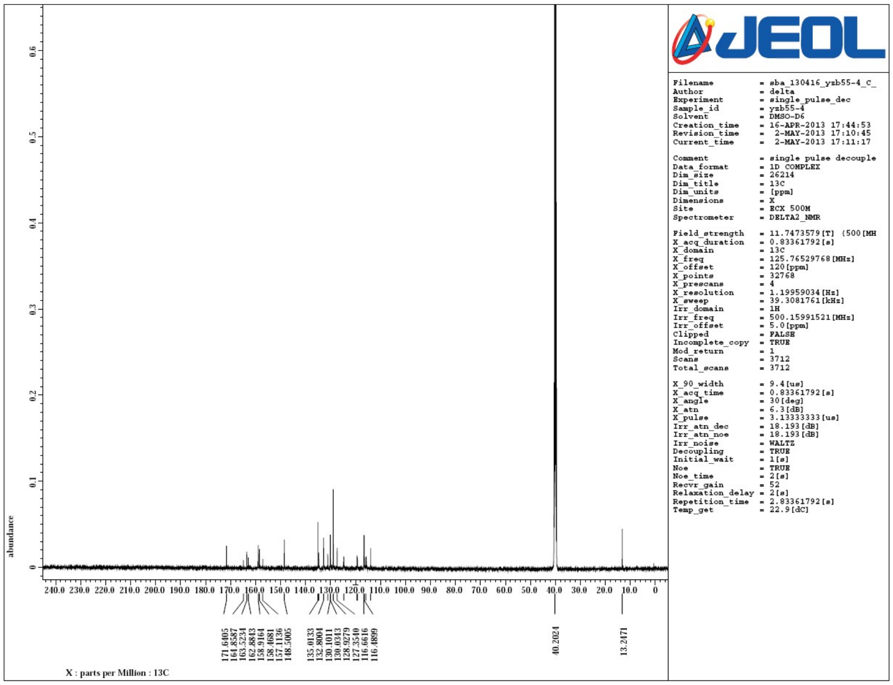

^{13}C NMR of Compound 7m

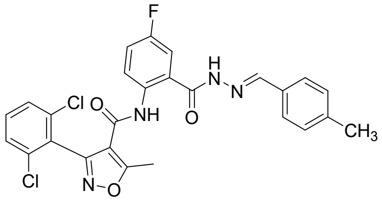

7n

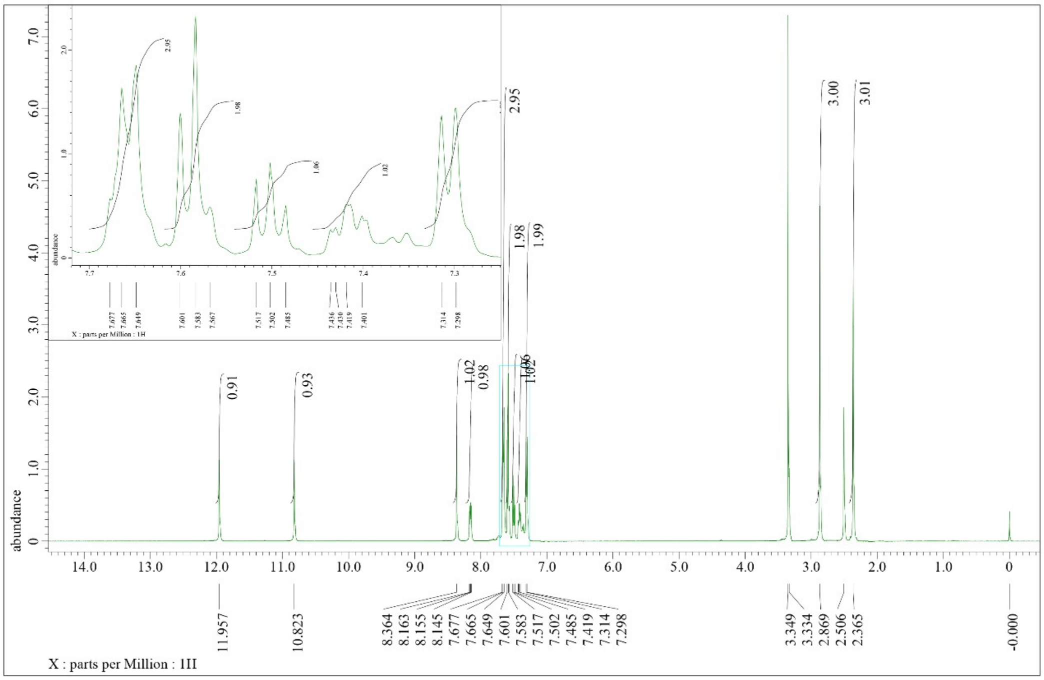

^{1}H NMR of Compound 7n

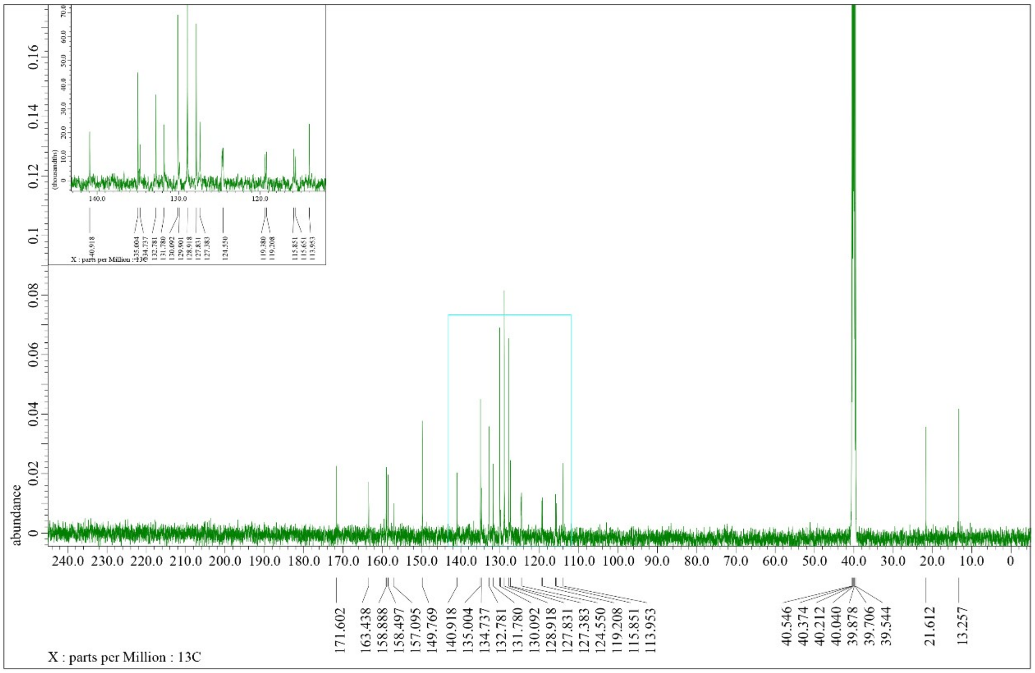

^{13}C NMR of Compound 7n

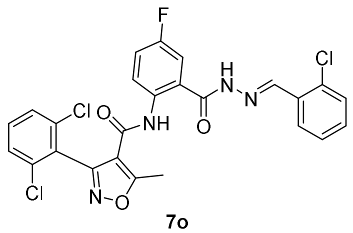

7o

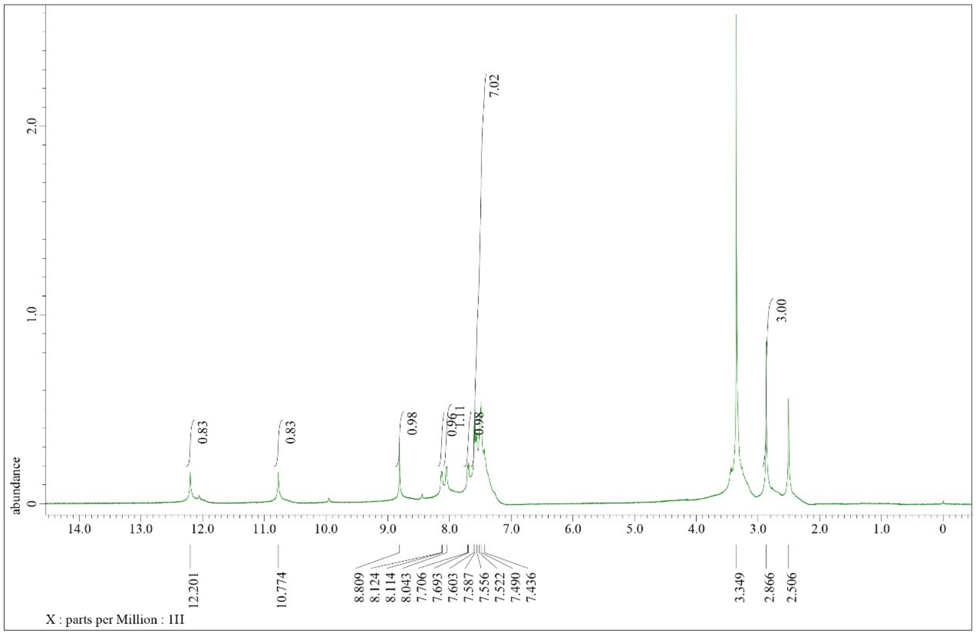

^{1}H NMR of Compound7o

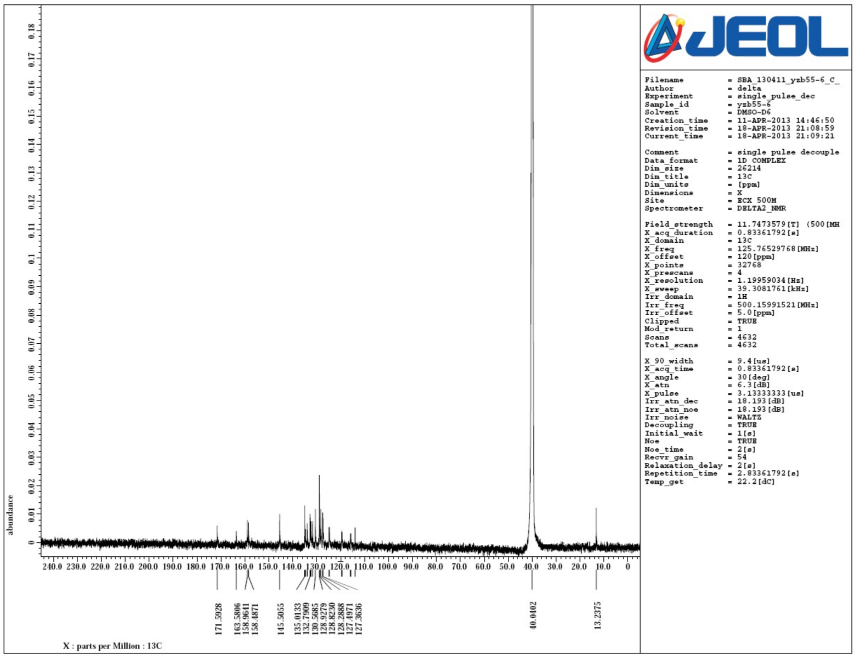

^{13}C NMR of Compound 7o

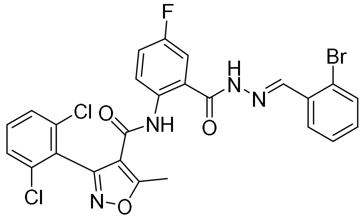

7p

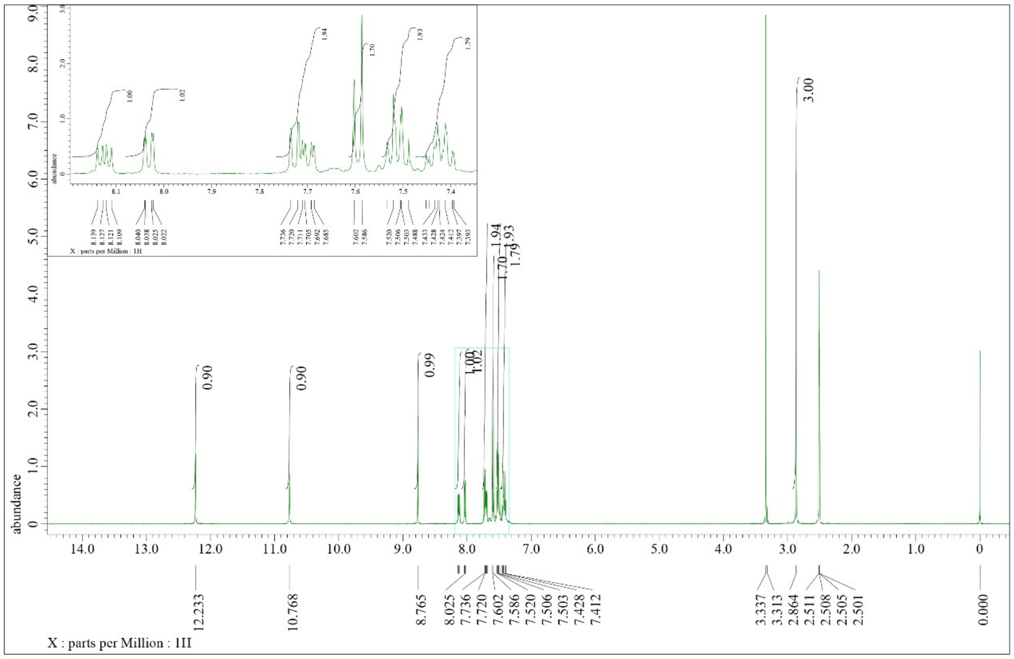

^{1}H NMR of Compound 7p

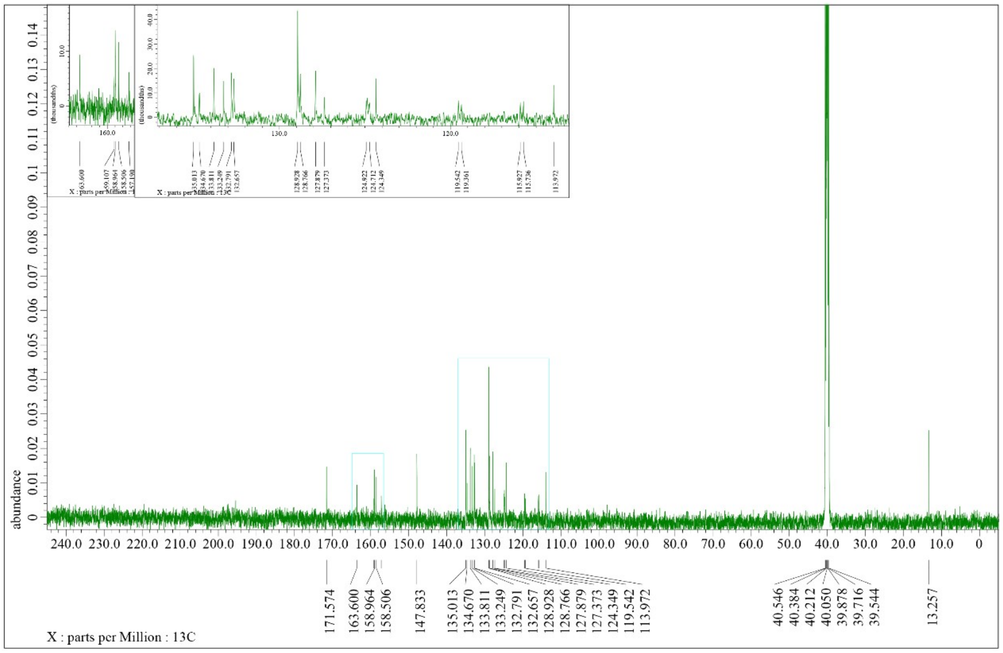

^{13}C NMR of Compound 7p

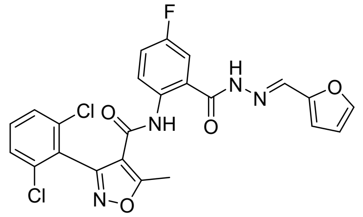

7q

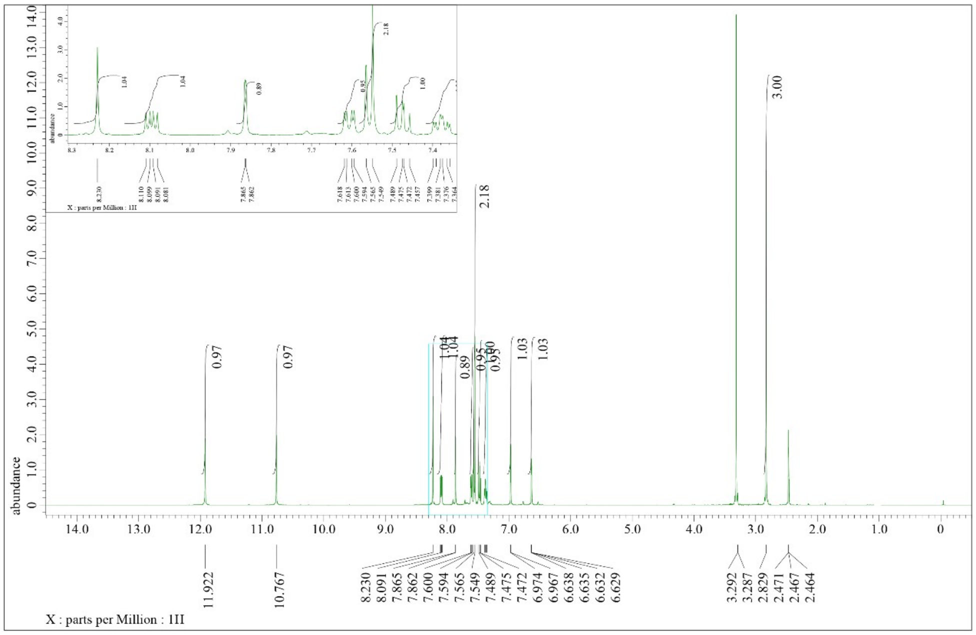

^{1}H NMR of Compound 7q

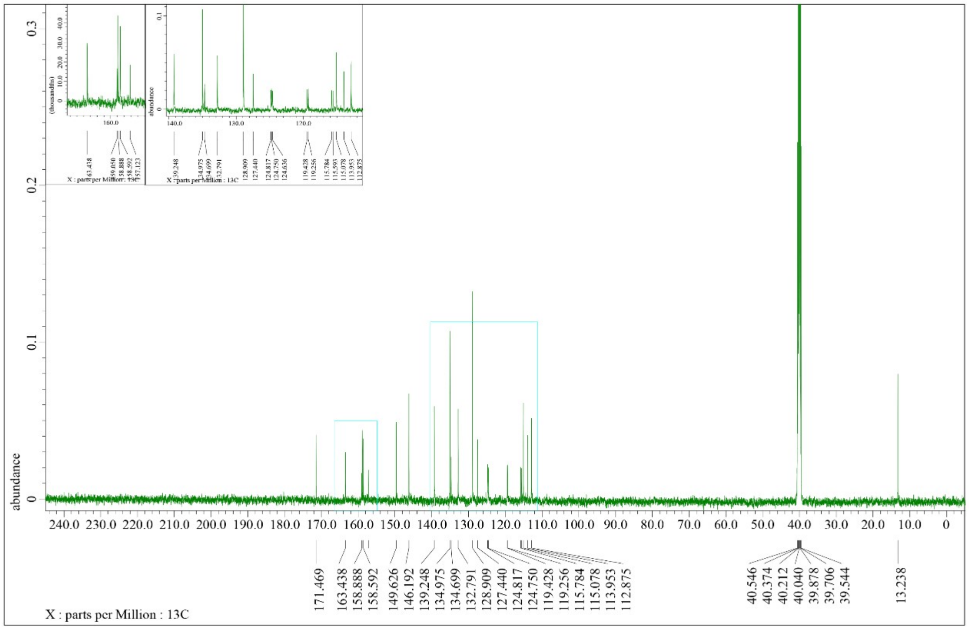

^{13}C NMR of Compound 7q

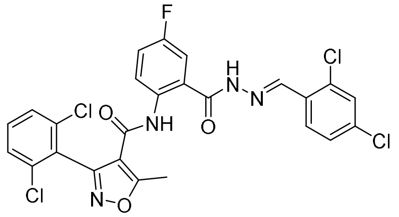

7r

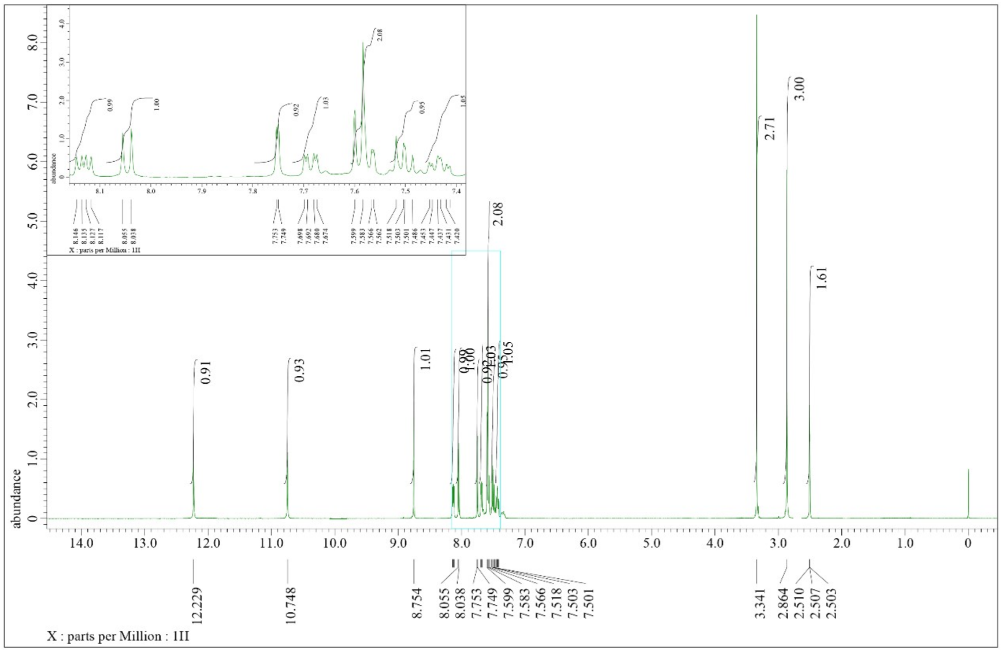

^{1}H NMR of Compound 7r

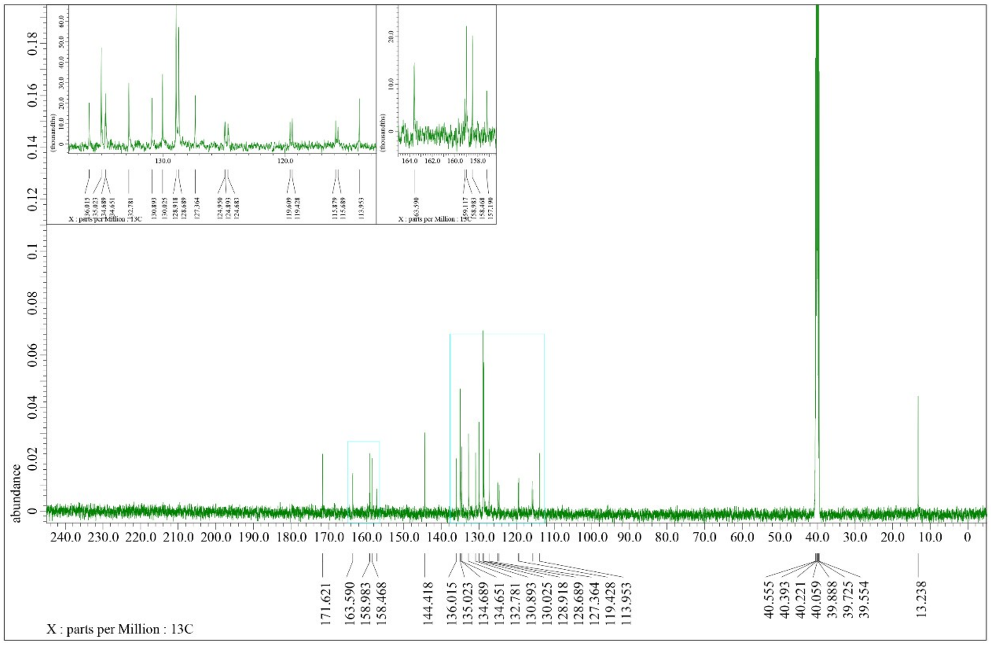

^{13}C NMR of Compound 7r

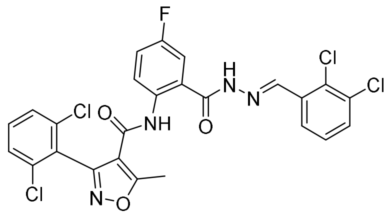

7s

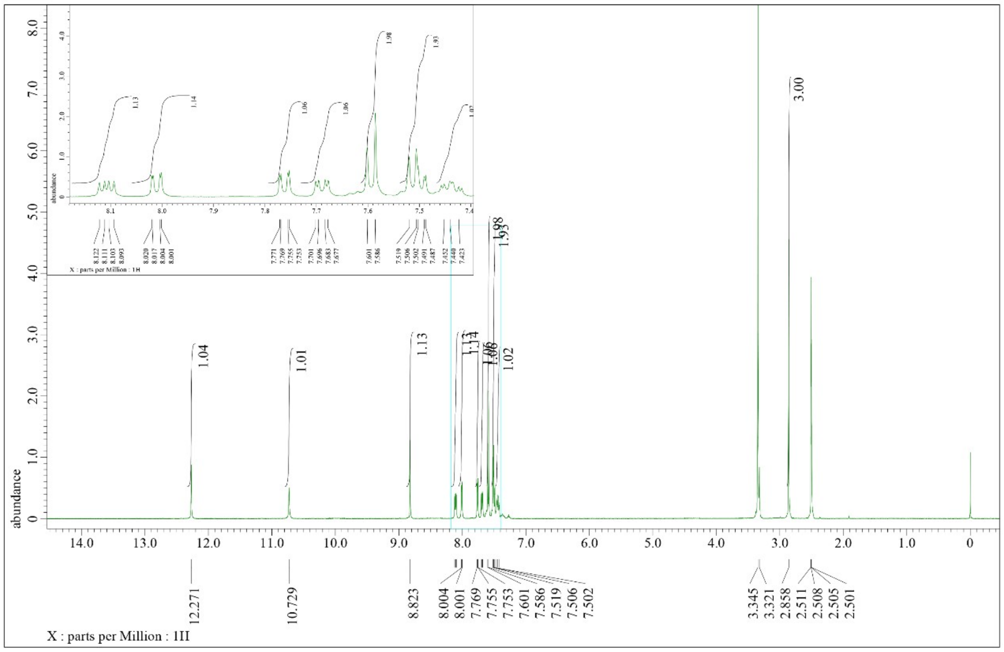

^{1}H NMR of Compound 7s

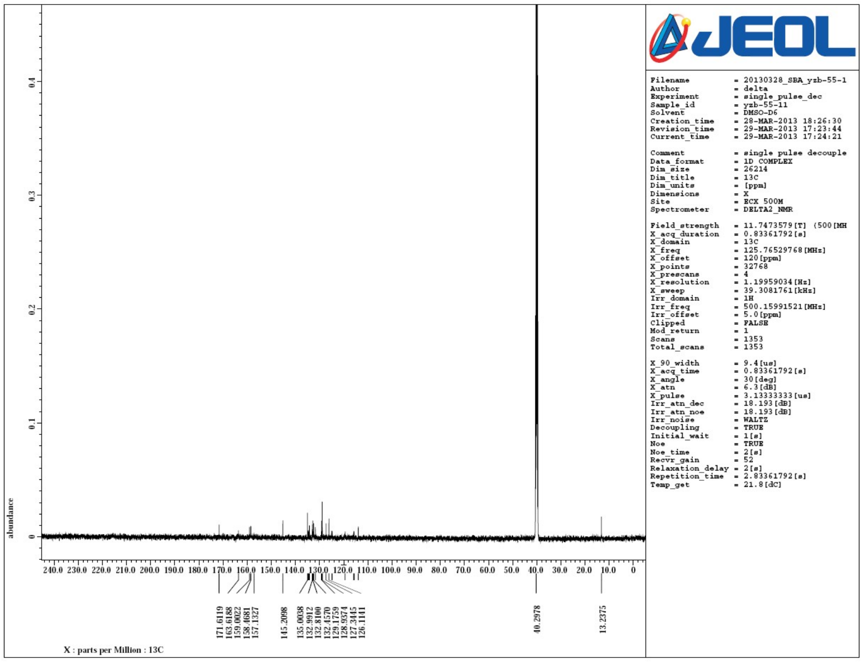

^{13}C NMR of Compound 7s

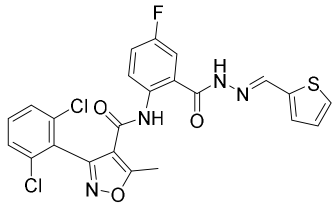

7t

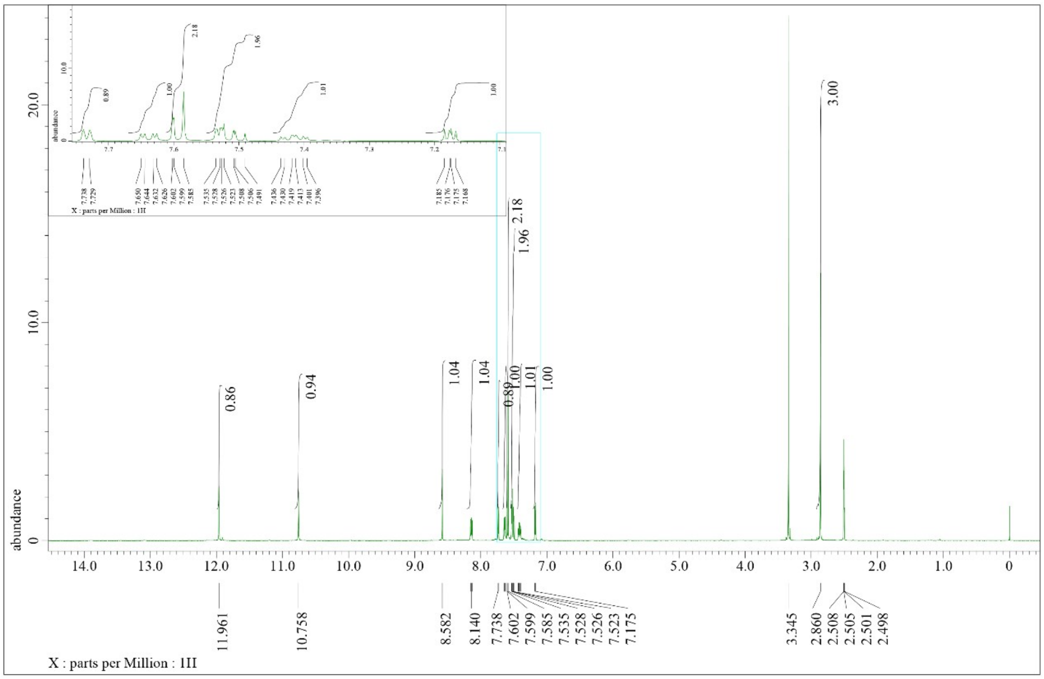

^{1}H NMR of Compound 7t

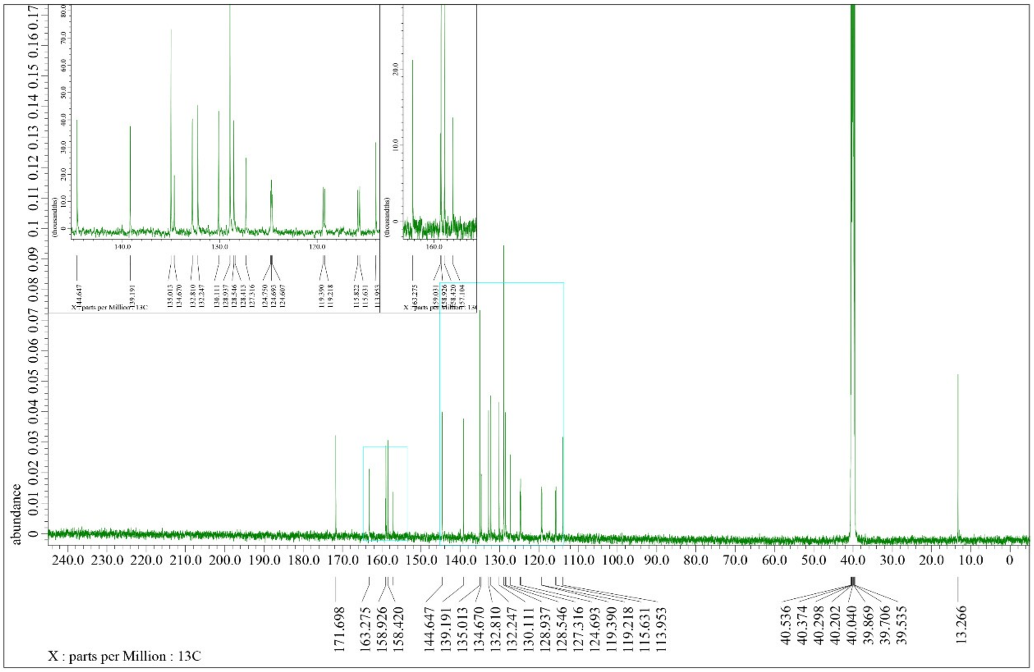

^{13}C NMR of Compound 7t

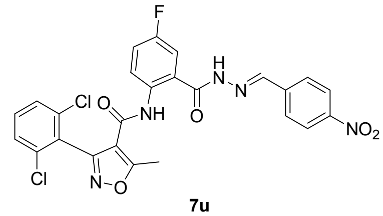

7u

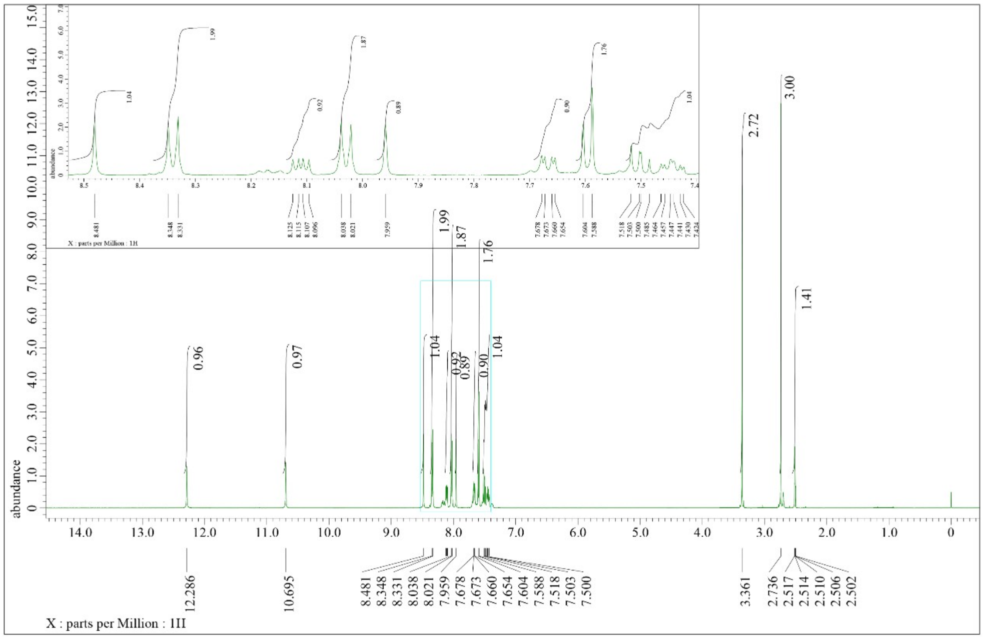

^{1}H NMR of Compound 7u

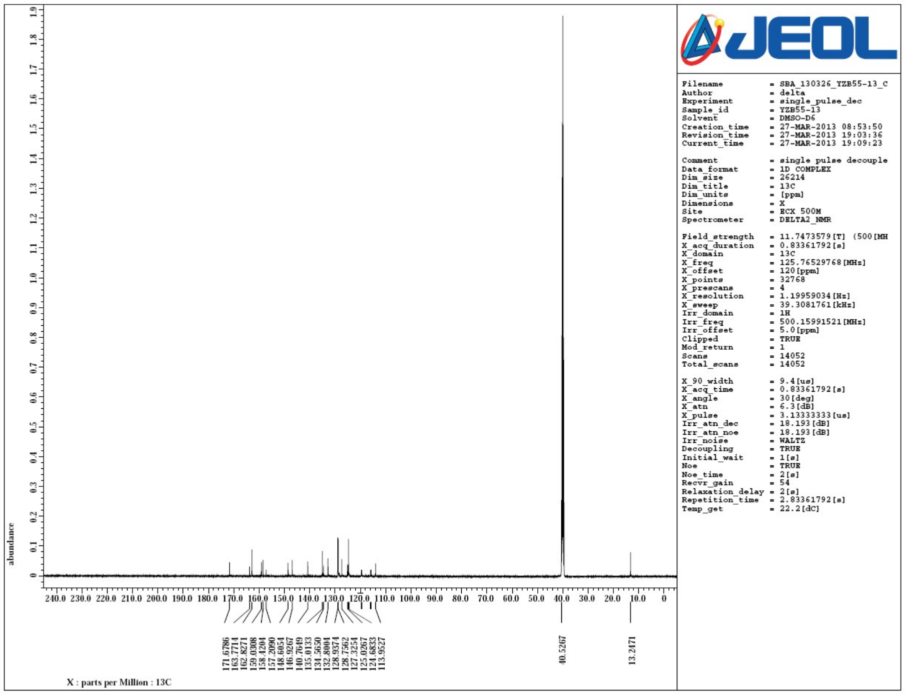

^{13}C NMR of Compound 7u

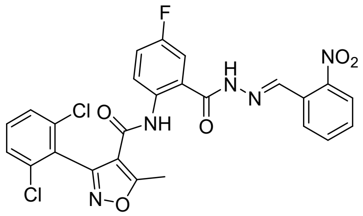

7v

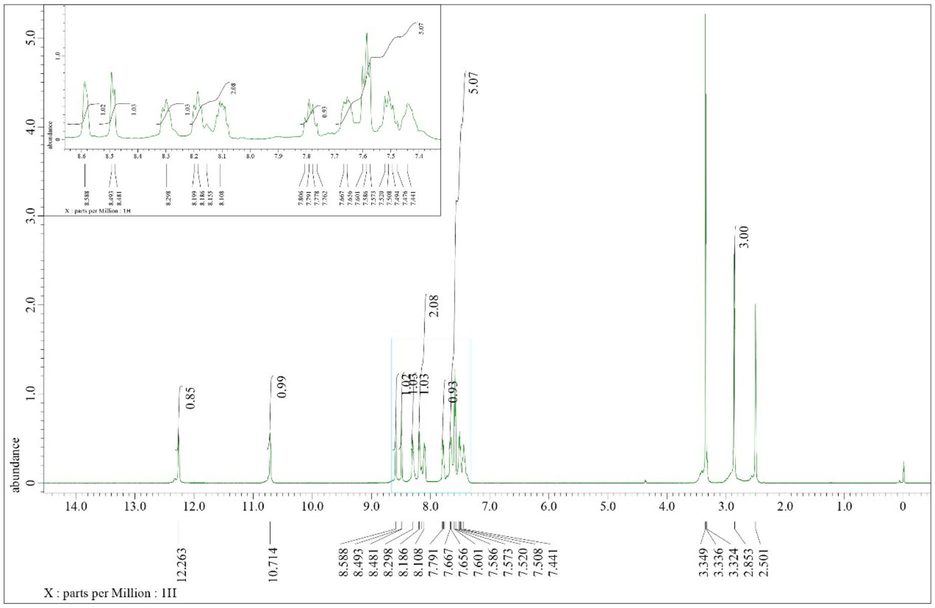

^{1}H NMR of Compound 7v

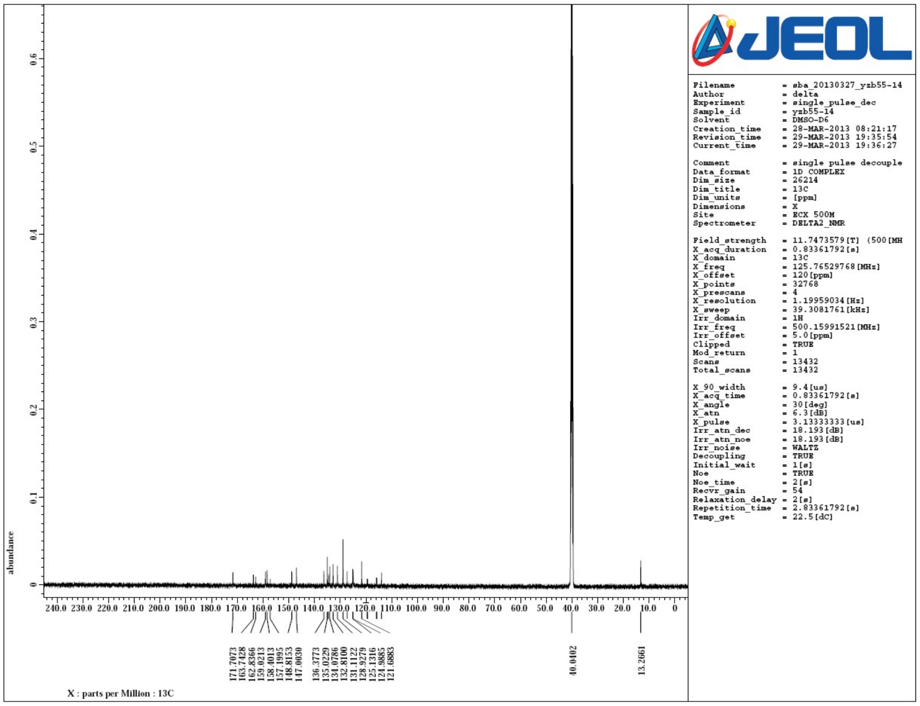

^{13}C NMR of Compound 7v
